# Proteomic analysis of sea urchin *(Strongylocentrotus purpuratus) *spicule matrix

**DOI:** 10.1186/1477-5956-8-33

**Published:** 2010-06-17

**Authors:** Karlheinz Mann, Fred H Wilt, Albert J Poustka

**Affiliations:** 1 Max-Planck-Institut für Biochemie, Abteilung Proteomics und Signaltransduktion, D-82152 Martinsried, Am Klopferspitz 18, Germany; 2 Department of Molecular and Cell Biology, University of California, Berkeley, CA94720-3200, USA; 3 Max-Planck-Institut für Molekulare Genetik, Evolution and Development Group, D-14195 Berlin, Ihnestrasse 73, Germany

## Abstract

**Background:**

The sea urchin embryo has been an important model organism in developmental biology for more than a century. This is due to its relatively simple construction, translucent appearance, and the possibility to follow the fate of individual cells as development to the pluteus larva proceeds. Because the larvae contain tiny calcitic skeletal elements, the spicules, they are also important model organisms for biomineralization research. Similar to other biominerals the spicule contains an organic matrix, which is thought to play an important role in its formation. However, only few spicule matrix proteins were identified previously.

**Results:**

Using mass spectrometry-based methods we have identified 231 proteins in the matrix of the *S. purpuratus *spicule matrix. Approximately two thirds of the identified proteins are either known or predicted to be extracellular proteins or transmembrane proteins with large ectodomains. The ectodomains may have been solubilized by partial proteolysis and subsequently integrated into the growing spicule. The most abundant protein of the spicule matrix is SM50. SM50-related proteins, SM30-related proteins, MSP130 and related proteins, matrix metalloproteases and carbonic anhydrase are among the most abundant components.

**Conclusions:**

The spicule matrix is a relatively complex mixture of proteins not only containing matrix-specific proteins with a function in matrix assembly or mineralization, but also: 1) proteins possibly important for the formation of the continuous membrane delineating the mineralization space; 2) proteins for secretory processes delivering proteinaceous or non-proteinaceous precursors; 3) or proteins reflecting signaling events at the cell/matrix interface. Comparison of the proteomes of different skeletal matrices allows prediction of proteins of general importance for mineralization in sea urchins, such as SM50, SM30-E, SM29 or MSP130. The comparisons also help point out putative tissue-specific proteins, such as tooth phosphodontin or specific spicule matrix metalloproteases of the MMP18/19 group. Furthermore, the direct sequence analysis of peptides by MS/MS validates many predicted genes and confirms the existence of the corresponding proteins.

## Background

The sea urchin embryo has been an important model organism in developmental biology for more than a century. This is due to the relatively simple construction of a translucent larva from about 1500 cells and the possibility to follow the fate of individual cells as development to the pluteus larva proceeds. Furthermore, sea urchins belong to the deuterostome lineage, which includes echinoderms and chordates. Thus, sea urchins are more closely related to vertebrates than they are to flies or worms, which provide other important model organisms, such as *Drosophila *or *Caenorhabditis*. Sea urchin pluteus larvae contain tiny calcitic skeletons, the spicules, which are produced by specialized skeletogenic cells, the primary mesenchyme cells (PMC). These cells fuse to yield a syncytical network that defines the geometry of the forming spicules [1,2]. Biomineralization takes place in a space confined by the PMCs and involves the secretion of organic precursors of a scaffold, the organic matrix, at least part of which eventually becomes incorporated into the growing biomineral [3,4]. The organic matrix makes up less than 1% of spicule mass and is composed of proteins and carbohydrates. It can be isolated from carefully cleaned spicules after demineralization with EDTA or dilute acid [5-7]. The first proteins identified in this matrix and characterized in some detail were a spicule matrix (SM) protein with a molecular mass of ~50 kDa, SM50 [8,9], and SM30 [10]. Using molecular biological and immunohistochemical methods several SM50-related spicule matrix proteins, PM27 [11], SM37 [12], SM29 and SM32 [13], were identified. It was suggested that they form a subfamily of SM proteins with alkaline isoelectric point, all containing in their sequence a C-type lectin-like domain (CTLLD) and a domain containing many short repeats of unusual amino acid composition [13]. SM30 turned out to be a member of another subfamily of SM proteins with CTLLD but acidic isoelectric point. Both SM50 and SM30 have been shown directly by immune-SEM techniques to be embedded in the mineral [14]. Screening of the recently published genome of *Strongylocentrotus purpuratus *[15] for C-type lectin-like domain-containing proteins suggested the presence of at least six members of the SM30 subfamily, SM30-A to F [16]. Computational methods, semi-quantative RT-PCR analysis and in situ hybridization studies implicated many more proteins in biomineralization events. This included previously un-described C-type lectin-like domain-containing proteins, at least six members of the extracellular and membrane-anchored mesenchyme specific MSP130 family, and one of 19 putative carbonic anhydrases encoded in the *S. purpuratus *genome [16]. In addition, several previous studies showed that various inhibitors interfere with spicule formation or elongation indicating that the affected enzymes could be involved in spiculogenesis. These were, for instance, the carbonic anhydrase inhibitor acetazolamide [17], inhibitors of metalloendoproteases [18,19], inhibitors of protein kinases [20-22], or the acetylcholinesterase inhibitor eserine [23]. 2 D electrophoresis of radiolabeled spicule matrix proteins indicated the presence of 45-50 proteins in the spicule matrix. However, only SM50 and two members of the SM30 subfamily were identified by Western blotting of the 2 D displays [24].

The sequencing of the *Strongylocentrotus purpuratus *genome [15] not only enabled sea urchin skeletal matrix analysis with computational and molecular biological methods, but also the direct analysis and identification of matrix proteins and their modifications by mass spectrometry-based high throughput methods [25-27]. Using such methods we have identified 231 proteins in the matrix of *S. purpuratus *spicules. The identifications validate many gene models and may explain some previous results obtained by molecular biological, immunohistochemical, and inhibitor studies. The identification of the protein repertoire of spicules also enables comparison between previously analyzed adult skeletal matrices and spicule matrix, defining a common toolkit of major biomineralizing proteins, but also allowing identification of compartment-specific components.

## Materials and methods

### Preparation of spicules

*S. purpuratus *embryos were raised in sea water containing 10 μg gentamycin/ml for 72-84 h at 15°C, then for 12-18 h at 4°C. Sodium azide was added to a final concentration of 0.1% and the embryos were allowed to settle at 4°C. Most of the medium was aspirated and the embryos were transferred to 50 ml tubes and centrifuged in a refrigerated table top centrifuge for 3 min at 1000 rpm. Spicules were isolated by adapting previously published protocols [24,28]. The embryos were washed in 5-10 volumes of cold de-ionized water and then in 5-10 volumes of cold 0.01 M Tris, pH 8. The final pellet was suspended in 10 volumes of 0.01 M Tris, pH 8, and homogenized using a Polytron type homogenizer at setting 6-7 for 1-1.5 min to break the embryos. Then SDS was added to a final concentration of 0.1% with swirling. The mixture was centrifuged in 50 ml tubes for 5 min at 4500 rpm in a refrigerated Eppendorf table top centrifuge, most of the dark purple supernatant was aspirated, and the dark purple pellet was suspended in a small volume of water. To this an equal volume of 4.5% sodium hypochlorite was added with swirling for 1-2 min before the mixture was centrifuged at 10000 g for 5 min in 2 ml tubes in an Eppendorf bench top centrifuge. The supernatant was removed and the now white pellet was suspended in calcium carbonate-saturated water, and 2-3 volumes of sodium hypochlorite were added. The mixture was swirled and immediately centrifuged at 10000 g for 5 min. The pellet was washed successively with calcium carbonate-saturated water, 100% ethanol and 100% acetone with interspersed centrifugation at 10000 g for 5 min. The final pellet was air-dried. The spicules were demineralized in 50% acetic acid (20 ml/g of spicule biominerals) at 4°C for 5-6 h with stirring. The turbid suspension was dialyzed successively against 2 × 100 vol. 10% and 2 × 100vol. 5% acetic acid at 4-6°C (Spectra/Por 6 dialysis membrane, molecular weight cut-off 1000; Spectrum Europe, Breda, The Netherlands). The slightly reddish precipitate, which formed during dialysis, was suspended in the clear supernatant and both were lyophilized together.

**Figure 1:**
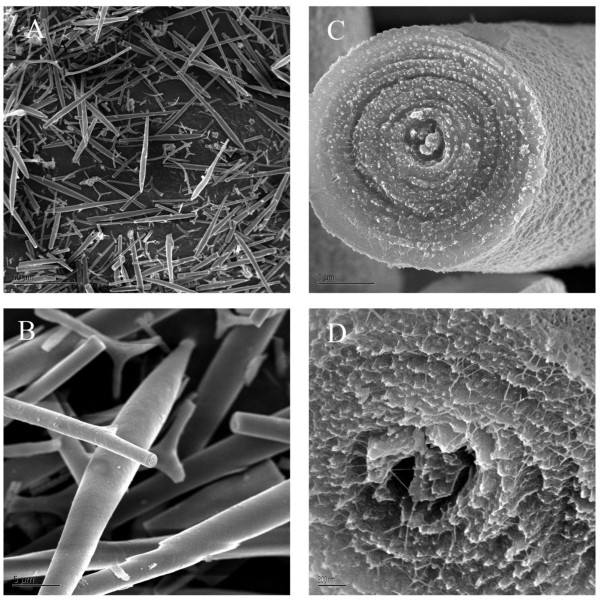
**Purified *S. purpuratus *spicules visualized by scanning electron microscopy**. A, section showing fragments of spicules prepared from pluteus larvae. Fragmentation is due to fractures caused by homogenization during purification. Maximal length of spicule fragments is 70-80 μm. B, higher magnification showing the clean surfaces of spicule fragments. C, cross-section of a fractured spicule with a diameter of 4 μm showing the concentric layers of mineral. D, deeper etching and higher magnification reveals presumed matrix fibers coursing through the mineral layers.

Characterization of spicules by SEM (Figure 1) was carried out exactly as previously described [14]. In brief, specimens were carbon coated (15 nm) before examination. Etching was carried out by exposure to pH6.0-6.5.

### Preparation of peptides

**Figure 2:**
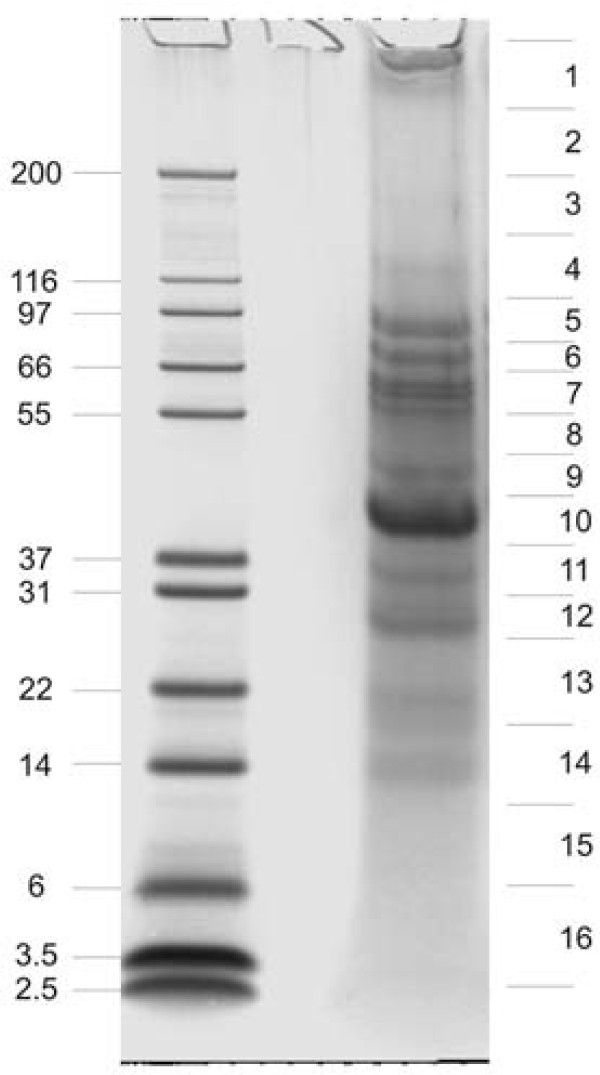
**PAGE separation of spicule matrix proteins**. The molecular weight of marker proteins is shown in kDa to the left. Sections excised for in-gel digestion are indicated to the right.

Matrix proteins were separated by SDS-PAGE in pre-cast 4-12% Novex Bis-Tris gels using the MES buffer system with reagents and protocols supplied by the manufacturer (Invitrogen, Carlsbad, CA). The kit sample buffer was modified by adding SDS and β-mercaptoethanol to a final concentration of 5% and 2%, respectively, and the sample was suspended in 40 μl sample buffer/200 μg of organic matrix, boiled for 5 min, cooled to room temperature and centrifuged. Part of the material remained insoluble and was removed by centrifugation. The supernatant was subjected to PAGE (40 μl/lane) and the separated proteins were stained with colloidal Coomassie (Invitrogen). Gels were cut into 16 slices (Figure 2), and identical slices of two lanes were used for in-gel digestion with trypsin [29] in each of three separate experiments. All slices were treated equally irrespective of staining intensity or presence of visible bands. The eluted peptides were cleaned with C18 StageTips before MS analysis [30].

### LC-MS and data analysis

C_18 _reversed phase LC and mass spectrometric analysis was performed using a Proxeon Easy-nLC (Proxeon Biosystems, Odense, Denmark; software version 2.0) coupled to a LTQ Orbitrap or LTQ Orbitrap Velos spectrometer (Thermo Fisher Scientific, Bremen, Germany) via a nanoelectrospray ion source (Proxeon Biosystems). All settings and procedures were almost identical to those described previously [31,32]. In short, full scans were recorded in the orbitrap at a resolution of 30,000 or 60,000 in LTQ Orbitrap Velos or 60,000 in LTQ Orbitrap (at *m/z *= 400) followed by CID-activated MS/MS of the ten most intense peptide ions in the LTQ analyzer. Data analysis was performed with MaxQuant v1.0.13.13 [33,34]http://www.maxquant.org/, a software package currently making use of the Mascot search engine (Matrix Science, London, UK; version 2.2.04) for database searches. The database consisted of the *S. purpuratus *predicted annotated gene models (Glean3) protein sequence database ftp://ftp.hgsc.bcm.tmc.edu/pub/data/Spurpuratus/fasta/Annotation[15], the corresponding reversed database, and the sequences of common contaminants including human keratins from IPIhuman. Identified Glean3 models were compared to the manually curated SPU models maintained in SpBase http://sugp.caltech.edu/SpBase/. Carbamidomethylation was set as fixed modification. Variable modifications were oxidation (M), N-acetyl (protein) and pyro-Glu/Gln (N-term). Initial peptide mass tolerance was set to 7 ppm and fragment mass was set to 0.5 Da. Two missed cleavages were allowed and the minimal length required for a peptide was seven amino acids. Two unique peptides were required for protein identifications. These could also be derived from different experimental sets. The peptide and protein false discovery rates (FDR) were set to 0.01. The maximal posterior error probability (PEP), which is the probability of each peptide to be a false hit considering identification score and peptide length [33,34], was set to 0.01. Only proteins identified in two of three experimental sets (replicates) were accepted. Identifications with only two unique peptides, or a protein PEP >1.0E-10, were manually validated considering the assignment of major peaks, occurrence of uninterrupted y- or b-ion series of at least 3 consecutive amino acids, preferred cleavages N-terminal to proline bonds, the possible presence of a2/b2 ion pairs and mass accuracy. The ProteinProspector MS-Product program http://prospector.ucsf.edu/ was used to calculate the theoretical masses of fragments of identified peptides for manual validation. BLAST analysis was performed with the program provided by NCBI http://www.ncbi.nlm.nih.gov/blast and searching against the non-redundant database for all organisms. Annotations were taken from Spbase entries http://www.spbase.org/SpBase/download/[35] when possible. FASTA and MPsrch search programs were used as provided by the European Bioinformatics Institute (EBI, http://www.ebi.ac.uk) searching against UniProt Knowledgebase and UniProtKB/Swiss-Prot protein sequence databases. Disordered protein structure was predicted using POODLE-I http://mbs.cbrc.jp/poodle/poodle-i.html[36] and subcellular localizations were predicted with Sherloc2 http://www-bs.informatik.uni-tuebingen.de/Services/SherLoc2[37]. Secretion signal sequences were predicted using SignalP http://www.cbs.dtu.dk/services/SignalP/[38] and non-classical secretion was predicted with SecretomeP http://www.cbs.dtu.dk/services/SecretomeP/[39]. Transmembrane helices were predicted using TMHMM http://www.cbs.dtu.dk/services/TMHMM/[40]. GPI anchoring was predicted with GPI-SOM http://gpi.unibe.ch/. Domains were predicted with NCBI Conserved Domain Search [41] and InterProScan http://www.ebi.ac.uk/Tools/InterProScan/[42] which, among others, also includes SignalP, TMHMM and Prosite. Sequence alignments were done with ClustalW2 http://www.ebi.ac.uk/Tools/clustalw2/index.html. Abundance of proteins was estimated by calculating the exponentially modified Protein Abundance Index (emPAI) [43] without retention time. Observed unique parent ions for this calculation were taken from MaxQuant Viewer/Identifications/Peptides/MS^2^-tables and comprised all unique ions with a mass of 700-2800 with different m/z for a given peptide, caused by different charge states and different variable modifications. Known and predicted signal peptides were not included as observable peptides in this calculation. Known and predicted transmembrane segments were also excluded if they constituted an important fraction of the whole sequence, such as in tetraspanin [Glean3:00884], where predicted accessible domains represented only ~50% of the entire sequence.

## Results and Discussion

Spicules prepared by the method used, which involves treatment with SDS and sodium hypochlorite, appear free of adherent extracellular material when viewed by electron microscopy [6,14] (Figure 1). Purified spicules were demineralized with acetic acid to obtain the intra-crystalline organic matrix. The organic matrix yield was approximately 0.007 mg/mg of spicules. Organic matrix protein separation by PAGE produced a protein band pattern (Figure 2) very similar to previously published ones [6,24]. Gels were sliced into 16 pieces (Figure 2), proteins were cleaved in-gel, and the resulting peptides were extracted and analyzed by mass spectrometry. The spicule matrix proteome comprised 218 proteins identified with high confidence (see Additional file 1: Spicule organic matrix proteins identified with high confidence). However, it is possible that some entries contain more than one protein sequence, while the sequences of other proteins may be distributed among several entries. Thirteen more proteins were identified tentatively (see Additional file 2: Tentatively identified spicule organic matrix proteins). Most of these 13 identifications were classified tentative because only one of the required minimum of two unique peptides satisfied manual validation criteria. Usual problems encountered included too many unexplainable major fragments, and lack of certain expected sequence specific characteristics, such as preferred cleavage in front of prolines, or mass errors >5 ppm. However, all of these proteins had PEP values < 1.E-4 and were therefore probably correct identifications. More detailed data on scores, distribution in gel slices, and other relevant protein and peptide information is tabulated in Additional files 3 and 4 (Additional file 3: Protein data; Additional file 4: Peptide data). The number of identified proteins from spicules is almost twice as high as that obtained from test, spine or tooth matrix [25,26]. This could be due to higher complexity of the spicule matrix, but could also be due to the use of a new generation of mass spectrometers (Orbitrap, Orbitrap Velos [31,32]) and software (MaxQuant, [33,34]). Thirty-eight percent of proteins (122 of 231) identified in spicule matrix were not reported in previous proteomic studies of sea urchin tooth, spine and test [25-27].

The identified proteins were classified as minor or major according to the calculated exponentially modified protein abundance index (emPAI [43]). However, despite the usefulness of emPAI for this purpose it should be clear that this is only an approximation to the real concentration of a protein in the spicule. The emPAI relates the number of theoretically possible peptides for a particular protein, obtained by *in silico *digestion of the database sequence, and the number of identified unique parent ions found for this protein. The abundance of a particular protein may therefore be affected by peptides not identified because of posttranslational modifications, wrongly assembled sequences in the database, or the occurrence of identical peptides in different protein sequences. In the following discussion the approximate 33% of proteins with the highest emPAI will be designated "high-abundance" or "major" protein.

Approximately one third of the identified proteins were known or predicted to occur in the extracellular space (see Additional files 1 and 2). Another third of identified proteins were known or predicted to be transmembrane proteins. These proteins usually contain large ectodomains that may have been incorporation into the matrix after shedding mediated by matrix metalloproteases (MMPs) or other proteases abundantly present in the mineralization space. To the best of our knowledge all peptides derived from such proteins were from known or predicted extracellular domains. Proteins associated with the Golgi or ER cellular compartments may have entered the mineralization space as by-products of secretion processes and may have been trapped by the growing mineral. Intracellular proteins, representing a minority in the spicule matrix proteome, may have entered the mineralization compartment by cell leakage or may be residual spicule surface contaminants that survived SDS treatment and bleaching.

In the following sections we discuss selected proteins and protein families of interest for biomineralization in general and sea urchin skeleton formation in particular. For a compilation of all proteins identified in proteomically analyzed skeletal matrices see Additional file 5: Compilation of proteins identified in *S. purpuratus *skeletal elements.

### Proteins containing a single C-type lectin-like domain

Sea urchin skeletal matrices contain a large group of proteins that contain as a common element a single C-type lectin-like domain (CTLLD). This similarity was first described for PM27 (primary mesenchyme cell-specific protein with an apparent molecular weight of 27 kDa) [11]. However, most members of this group are labeled SM (spicule matrix) protein with addition of the molecular weight, following the nomenclature introduced for the first spicule matrix proteins characterized by cDNA-derived amino acid sequence, SM50 [8,9] and SM30 [10]. In addition to the CTLLD many of these proteins contain in their sequence a stretch of short repeats of the type Pro-X-Y with X most frequently Asn, Gln, Gly or His. The number of repeats and their location, N-terminal or C-terminal to the C-type lectin-like domain, varies in different proteins [16]. The proteins were attributed to two families, the SM30 family and the SM50 family, according to the sequence similarities of the C-type lectin-like domains (see Additional file 6: Alignment of CTLLD sequences of SM proteins).

#### The SM30 family

**Figure 3:**
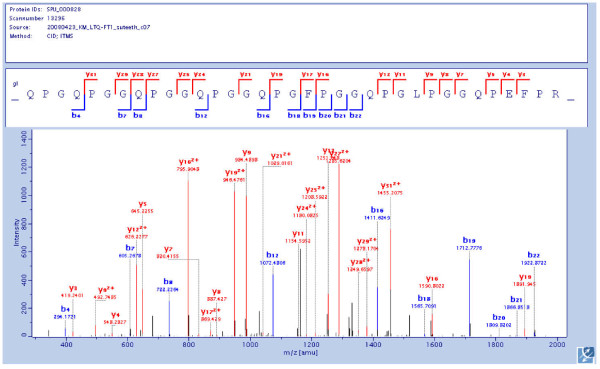
**MS/MS spectrum of a SM30-D peptide**. This spectrum shows the MS/MS analysis of a triply charged peptide confirming the annotated predicted sequence contained in SPU:000828. As to be expected almost all of the major y-ions correspond to preferential cleavages N-terminal to prolines. The N-terminal Gln is cyclized to pyroglutamine. The mass error was 0.11 ppm, the PEP score was 3.01E-46 and the Mascot score was 63. Spectra of other unique peptides are shown in additional file 6.

This family consists of the six conceptual gene products SM30A-F, to which the sequence contained in Glean3:00164 (Sp-Clect) may be added. Blast search against the non-redundant NCBI protein database matched this entry to a predicted protein "similar to SM30" (LOC581461/XP_786548.1/XP_001188111), in agreement with sequence comparison (see Additional file 6). Recently the SM30A-F gene expression patterns were determined in adult and embryonic biomineralized tissues of *S. purpuratus *[44], and a comparison to proteomic data is shown in the upper part of Table 1. However, the results may not be entirely comparable because proteins for proteomic analyses were derived from hypochlorite-washed biomineral, while the mRNA for RT-PCR analysis was isolated from tissues producing the mineralized structures. The most similar putative transcription products of this group are SM30A, B and C with sequence similarities between 93 and 99%. In fact it may be difficult to discern these three predicted proteins by most experimental techniques. We identified with high confidence two peptides (see Additional file 4) matching both, SM30B and SM30C in the spicule matrix. SM30-A was not detected. None of the three predicted proteins was identified in an adult skeletal matrix [25-27] (see Additional file 5). In contrast, transcription of SM30A gene was shown in spicule-related tissues andSM30B/C message was detected in all examined tissues [44]. Concordantly, neither SM30-D protein nor messenger RNA was detected in spicule or spicule-related tissues. However, SM30-D mRNA was detected in tissues related to spine, test and tooth [44], while SM30-D was not detected by previous proteomic analysis of the mineral matrix [25-27]. Re-analysis of old raw-files with the new MaxQuant software identified traces of SM30-D in all adult skeletal elements. The data were, however, not sufficient for positive identification. Searching for the causes of our failure to identify SM30-D in adult tissues, we noticed that the SM30-D protein sequences in our database and SpBase were not the same. The major differences were in the N-terminal third, which contains the Pro-rich repetitive sequences (see Additional file 7). Re-analysis of old raw-files against a database containing both sequence variants identified more peptides (Figure 3; Additional file 7) than before and confirmed the sequence contained in the SpBase (SPU:000828) entry. In agreement with expression analysis data, SM30-D was now identified in all adult skeletal matrices (Table 1). SM30-E was highly abundant in adult skeleton and in spicule matrix. This was in reasonable agreement with RT-PCR expression analysis results and indicates a major role for this protein in sea urchin mineralization in general. SM30-F was not identified in spicule proteome or transcriptome, but occurred in some adult tissues (Table 1).

**Table 1:** Occurrence of SM proteins and other C-type lectin-like domain-containing proteins in sea urchin skeletal element proteomes

Protein	Database entry	Test ^4^	Spine ^4^	Tooth ^5^	Spicule
**SM30-A**	**Glean3:00825**	- [**-**]	- [**-**]	- [**-**]	- [**++**]
**SM30-B/C ^1^**	**Glean3:00826** **Glean3:00827**	- [**+**]	- [**++**]	- [**+**]	+ [**++**]
**SM30-D ^3^**	**Glean3:00828** **SPU:000828**	+ [**++**]	++ [**++**]	++ [**++**]	- [**-**]
**SM30-E**	**Glean3:04867**	++ [**++**]	++ [**+**]	++ [**++**]	++ [**+**]
**SM30-F**	**Glean3:04869**	- [**-**]	+ [**+**]	- [**+**]	- [**-**]
**Sp-Clect**	**Glean3:00164**	-	(+)	+	++
*SM50*	*Glean:18811*	++	++	++	++
*PM27 ^3 ^*	*Glean3:30147* *UniProtKB:Q26616*	++	++	++	++
*SM29*	*Glean3:05990*	++	++	++	++
*Sp-Clect_14/similar to SM29*	*Glean3:05991*	(+)	++	++	++
*Sp-Adndatrnas/similar to SM29*	*Glean3:05992*	+	-	+	-
*SM32*	*Glean3:18810*	++	++	++	++
*SM37*	*Glean3:18813*	++	++	++	++
*Sp-Clect_13*	*Glean3:05989*	++	++	++	++
*Sp-Clect_25*	*Glean3:11163*	++	++	(+)	++
Sp-C-lectin/ PMC1	Glean3:27906	+	+	+	+
Sp-Clect_76	Glean3:13825	++	+	++	++

**^++^**among the 33% of most abundant proteins in the respective skeletal element; **+**, present; **(+)**, tentatively identified; **-**, not identified. **^1^**, SM30-B and SM30-C are 97% identical, two identified peptides potentially occur in both proteins. **^2^**, it was noticed during the present data analysis that SpBase and Glean3 database contained different sequences for SM30-D; re-analysis confirmed the presence of SM30-D in adult tissues**.^3^**, the absence of the PM27 sequence in the Glean3 database was only noticed, and its sequence added, during data evaluation for the present report. The data for test, spine and tooth were obtained by re-analysis of the old raw-files with MaxQuant. **^4^**, [25,27]. **^5^**, [26,27]. Recent gene expression data for the SM30 family [derived from Figure 2 and Figure 5 of reference [44]] are shown in square brackets. The SM30 family of proteins is in bold print; the SM50 family of proteins is in italics.

#### The SM50 family

This group is more heterogeneous than the SM30 family. The first member of this family characterized by cDNA sequence analysis, SM50 [8,9], was the most abundant protein in all analyzed skeletal elements ([25,26]; Additional file 1; Additional file 5). Also among the most abundant proteins in all matrices were the closest relative of SM50, SM32, and SM29. Other SM proteins identified in all skeletal matrices with high abundance were SM37, PM27 and Sp-Clect_13 [Glean3:05989]. Sp-Clect_14/similar to SM29 [Glean3:05991] was identified at high abundance in all skeletal matrices except test matrix. Sp-Clect_13 and Sp-Clect_14 were identified previously as putative matrix proteins and their corresponding genes are located next to the gene coding for SM29 [16]. Finally, Sp-Clect_25 [Glean3:11163] was tentatively grouped with the SM50 family because the sequence of its CTLLD was more similar to the SM50 family than to the SM30 family or other proteins in Table 1 (see Additional file 6). Sp-Clect_25 was a major protein of spicule, test and spine matrix but was only tentatively identified in tooth matrix (Table 1).

#### Other C-type lectin-like matrix proteins

Two proteins containing a single CTLLD, and apparently belonging to neither of the above families, were Sp-C-lectin/PMC1 [Glean3:27906] and Sp_Clect_76 [Glean3:13825]. Sp-C-lectin/PMC1 has been suggested previously to be a spicule matrix protein [16]. This protein was present in the matrices of all four analyzed skeletal elements, but did not belong to the major proteins. In contrast Sp-Clect_76 was a major protein of test, tooth and spicule matrix, but was of moderate abundance in spine matrix. The Sp-Clect_76 sequence does not include a predicted signal sequence. It does, however, contain a proline-rich repeat domain C-terminal to the CTLLD. The CTLLD of Sp_Clect_76 contained only 3 cysteines instead of the usual pattern of at least four conserved Cys. This may indicate that the sequence is not complete.

### Metalloproteases and other proteases

Sea urchin primary mesenchymal cells and embryos cultured in the presence of metalloprotease inhibitors show inhibition of spiculogenesis [18,19,45]. If inhibitor was added to embryos that had already formed a small tri-radial skeleton, further elongation of spicules was blocked. An obvious role for MMPs in spiculogenesis in particular, and skeletogenesis in general, would be the proteolytic processing of matrix molecule precursors. In fact SM30-B is cleaved to produce a lighter species upon incorporation into the growing spicule [46]. MMPs may also be supposed to play a role in metamorphosis, growth, and repair events. Proteomic analysis shows that the matrix of skeletal elements contains several MMPs at high abundance, indicating that these proteases were active soluble constituents of the membrane-confined mineralization compartment before they eventually became incorporated into the growing mineral [25,26]. The spicule matrix contained many of the MMPs already identified in spine, test and tooth matrix (see Additional file 1), but also MMPs not previously identified in sea urchin mineral matrices. The most abundant MMPs were a group of four related proteins (Sp-Mmp18/19-like 3-6) encoded in Glean3:05723, Glean3:05577, Glean3:09924 and Glean3:09925 (see Additional file 8). Overlapping, but different, peptides validated three of these four sequences (see Additional file 8) but sequence coverage by MS/MS-sequenced peptides was not high enough to decide whether the complete sequences were different gene products, splice variants, or sequencing and assembly errors. The distribution of various MMPs in all analyzed skeletal elements is shown in Table 2. The spicule matrix also contained a highly abundant protease, Sp-Unk_38 [Glean3:07355], and several less abundant proteases, which were not reported to be matrix proteins previously (see Additional file 1), such as the trypsin-like Sp-TrypL [Glean3:00049], the presumptive aminopeptidase Sp-ApdpL [Glean3:00619] and Sp-Adam/TS16/18L [Glean3:21193]. In striking contrast to the high number and abundance of proteases was the scarcity of protease inhibitors. These were Sp-Timp3b and possibly two or three α-macroglobulin-like proteins encoded in [Glean3:24564], [Glean3:24565] and [Glean3:05193]. This imbalance may indicate that the mineralization spaces delineated by PMCs or their descendants are compartments of high proteolytic activity and underline the importance of proteolysis in matrix assembly.

**Table 2:** Distribution of matrix metalloproteases in different matrices

Glean3_entry	Sp-base name	Test ^1^	Spine ^1^	Tooth ^2^	Spicule
**05385**	Sp-Mt5/MmpL3	+	-	+	-
**05577**	Sp-Mmp18/19L3	-	-	-	++
**05723**	Sp-Mmp18/19L6	-	-	-	+
**09924**	Sp-Mmp18/19L5	-	-	-	++
**09925**	Sp-Mmp18/19L4	-	-	-	++
**12549**	Sp-Mt1-4/MmpL4	+	-	-	-
**13669**	Sp-Mt1-4/MmpL5	+	+	++	++
**13670**	Sp-Mt1-4/MmpL6	++	++	++	++
**28748**	Sp-Mt1-4/MmpL7	+	+	+	++
**28749**	Sp-Mt5/MmpL2	++	++	++	++

Entries are ordered with decreasing Glean3 numbers**. ^1^**, [25,27]. **^2^**, [26,27]. **++**, among the 33% of most abundant proteins in the respective skeletal element; **+**, present; **-**, not identified.

### Other proteins of importance to sea urchin skeletogenesis

#### Carbonic anhydrases

A key enzyme in biomineralization events involving calcium carbonate is carbonic anhydrase which catalyzes the reversible conversion of carbon dioxide to protons and bicarbonate. Bicarbonate in turn precipitates with calcium ions to form calcium carbonate and protons. The only carbonic anhydrase of spicule matrix is Sp-Cara7LA [Glean3:12518]. This is the most abundant carbonic anhydrase in all four proteomically analyzed skeletal matrices, indicating that the presence of this extracellular enzyme is an absolute requirement for mineralization in sea urchins. Only tooth matrix contains a second, highly abundant carbonic anhydrase, Sp-Cara12LB [Glean3:25722] [26] (see Additional file 5).

#### MSP130-related proteins

A monoclonal antibody to a 130 kDa cell surface glycoprotein was previously shown to inhibit calcium accumulation and skeleton formation in cultured embryonic sea urchin cells [47]. Subsequent studies indicated that this protein, MSP130, was produced specifically by skeletogenic primary mesenchyme cells [48] and was attached to the outer cell surface by a phospholipase-sensitive lipid anchor [49]. Subsequent studies identified two MSP130-related proteins [13], and finally analysis of the *S. purpuratus *genome revealed the presence of four new members of this family resulting in a total of seven members [16]. The original MSP130 sequence ([49]; [UniProtKB:P08472]) is almost identical to the sequence contained in Glean3:13821. The major difference is an insert of 26 amino acids between position 721 and 722 of the original sequence. This insert was completely covered by MS/MS-derived peptide sequences, thus confirming the Glean3 sequence. Glean3:02088 contained an N-terminally truncated sequence of MSP130. Entries Glean3:06387 (Sp-Msp130L) and Glean3:13823 (Sp-Msp130r3) also share extended regions of sequence identity, but a few unique MS/MS-sequenced peptides occurring in each of them may indicate different gene products and some micro-heterogeneity between individuals (see Additional file 4: Peptide data). Finally, Gleane3:16506 (Sp-Msp130r2) and Glean3:21385 (Sp-ApL) apparently code for different, but overlapping, regions of the MSP130r2 sequence. Several of the MSP130 family members are highly abundant in all analyzed matrices (Table 3). Others, like MSP130r5, have a more limited expression. The only family member that was not identified in any matrix was MSP130r6 (Table 3).

**Table 3:** Occurrence of MSP130 and related proteins in *S. purpuratus *skeletal elements

Glean3_entry	Sp-base name	Test ^1^	Spine ^1^	Tooth ^2^	Spicule
**06387**	Sp-Msp130L	+	+	++	++
**13821** **02088**	Sp-Msp130_1 Sp-Msp130	++	++	++	++
**13822**	Sp-Msp130r1	++	++	+	++
**13823**	Sp-Msp130r3	++	++	+	++
**14492**	Sp-Msp130r6	-	-	-	-
**14496**	Sp-Msp130r4	+	+	-	+
**15763**	Sp-Msp130r5	-	-	-	+
**16506** **21385 **^3^	Sp-Msp130r2 Sp-Apl	++	++	++	++

Entries are ordered with decreasing Glean3 numbers. **^1^**, [25,27]. **^2^**, [26,27]. **^3^**, Sp-ApL (alkaline phosphatase-like) has only limited similarity to alkaline phosphatases but is very similar to MSP130-related 2. **++**, among the 33% of most abundant proteins in the respective skeletal element; **+**, present; **-**, not identified.

#### Acetylcholine esterase

The appearance of acetylcholine esterase in spicule matrix may be somewhat surprising. However, the acetylcholine esterase inhibitor serine was reported to inhibit spiculogenesis in cultured micromeres and spicule elongation in embryos [23]. The enzyme was identified as a major protein (see Additional file 1) in spicule matrix exclusively, indicating that acetylcholine, or rather its removal, plays a role in the regulation of spicule formation, but apparently not the formation of other mineralized structures.

#### Immunophilins

The immunophilins are a group of proteins including cyclophilins and FK506-binding proteins with peptidylprolyl cis-trans isomerase activity. They catalyze the conversion of cis to trans at Xaa-Pro bonds in proteins and act as chaperones [50]. The cyclophilins have also been implicated in sea urchin spiculogenesis [51]. The cyclophilin encoded in Glean3:07484 (cyclophilins 1) is specifically expressed in skeletogenic PMCs in the embryo [51,16]. This protein was identified among the major proteins of spicule (Additional file 1) and tooth matrix [26], but was not found in test and spine matrix. An FK-binding protein (Sp-Fk506bp2 [Glean3:18964]) was detected at lower abundance in spicule matrix (see Additional file 1) but was a major protein in tooth matrix [26] (Additional file 5). The exact role of immunophilins in skeletogenesis is unknown at present.

#### Miscellaneous

Glean3:18406 (Sp-Hypp_2998) and Glean3:18407 (Sp_Hypp_2999) contain almost identical sequences of proteins with a mass of 36 kDa and a calculated pI of 11, and belong to the most abundant proteins in all analyzed matrices (see additional files 1 and 5). The major difference between these two sequences is in the first 20 amino acids. The gene(s) coding for them is immediately adjacent to the gene coding for Sp-P16 [16], a regulator of spiculogenesis [52]. P16 was not identified in matrices. However, this could be due to a lack of tryptic cleavage sites in the sequence. The sequences of Sp-Hypp_2998 and 2999 contain 30% glycine and 10% proline. A clear secretion signal is predicted for Sp-Hypp_18407, but not for Sp-Hypp_18406. A C-terminal predicted transmembrane signal is identical for both entries (amino acids 337-357). Secondary structure predictions indicated random coil for most of the sequence in agreement with a POODLE-I prediction of disordered structure. The lack of any significant database match prevents any prediction of a function.

### Other proteins involved in mineralization events

Several Glean3 entries validated by peptide sequences in the present study contain one or more annexin repeats or are similar to annexins of other species (Additional file 1: spicule organic matrix proteins). Annexins are a family of Ca^2+^- and phospholipid-binding proteins, occurring intracellularly and extracellularly, implicated in cell proliferation and differentiation by binding to cytoskeletal components, ion channels, and extracellular matrix [53-55]. Annexins A2, A5 and A6 are membrane-bound components of mammalian osteoblasts and chondrocytes matrix vesicles, the organelles where mineralization is initiated [56]. Therefore the annexins and annexin-like proteins contained in the spicule matrix, and other skeletal matrices, may play a role in sea urchin mineral formation, too. Entries Glean3:07441 and Glean3:14160 encode annexin domain-containing proteins of high abundance in spicule matrix, but not in other matrices. Less abundant spicule matrix annexins were Glean3:21041 and Glean3:26001. Other annexins of high abundance in spicule matrix, Sp-Anxn(_1) [Glean3:11106/11107] and Sp-Anxa7_2 [Glean3:25962/00475], also occur at lower abundance in spine and tooth matrix (see Additional file 5). Another family of Ca-dependent phospholipid-binding proteins, the copines, was also present in spicule matrix. Copine A [Glean3:09516] is a major protein and copine D/L [Glean3:20636/08019] is a minor spicule matrix protein (see Additional files 1 and 5). Copines appear to be involved in membrane trafficking and regulatory events, and the high abundance of copine A may indicate a role in spiculogenesis.

Thrombospondins play a role in vertebrate skeleton mineralization [57]. The spicule matrix contained two thrombospondins among the major proteins (see Additional file 1). These were Sp-Thsd7b [Glean3:19739] and Sp-Thsd7b_2 [Glean3_25454]. Both sequences contained unique peptides but also share common peptides (see Additional file 9). Alignment of the sequences showed a nucleus of almost identical sequences but differences in the N-terminus. Furthermore, the C-terminal half of Sp-Thsd7b was without match in Sp-Thsd7b_2. Our data did not allow deciding unequivocally whether both putative proteins are in fact different gene products or just one protein, with a few micro-heterogeneities, spread over two faulty entries. Another thrombospondin, Sp-Thsd4_1 [Glean3_00204/26040] is specific for tooth matrix [26] (see Additional file 5).

## Conclusions

Similar to the data obtained by proteomic analysis of a few other biomineral matrices, such as chicken eggshell matrix [58] and other sea urchin skeletal matrices [25,26], the *S. purpuratus *spicule matrix turned out to be more complex than previously anticipated. However, the presence of more than 40 spots in a 2 D electrophoretic separation of spicule matrix proteins [24] suggested that there were more than the few then known SM proteins in this matrix. Of course not all of the more than 200 proteins identified in the present report may be supposed to play a role in matrix architecture or its assembly. For instance, the list (see Additional file 1) contains many ER and Golgi apparatus residents that may have reached the mineralization space as by-products of the secretion of specific matrix proteins. Still others, such as the many transmembrane proteins, have extended ectodomains that may have been cleaved off by the many proteases present in the mineralization space and may reflect the composition of the extracellular surface of the cells delineating the mineralization cavity. Still other proteins, such as BMP and several kinases, may testify to regulatory events involved in spiculogenesis. This clearly demonstrates that there is no mechanism to exclude non-specific proteins. Each protein that has reached the mineralization space will be incorporated into the growing mineral. This of course renders it difficult to discern specific matrix proteins, or components involved in matrix assembly, from un-intentionally trapped non-specific components. Probably the candidates for specific matrix components may be searched most safely among the major proteins. A comparison between the components of all analyzed matrices (see Additional file 5) indicates that proteins such as SM30-E, SM50, SM29, SM32, SM37, PM27, or Sp-Clect_13, which are among the most abundant components of all analyzed matrices, may be indispensable for every kind of mineralization event in the sea urchin. SM50 is the only matrix protein that has been shown experimentally, by use of morpholino antisense oligonucleotides, to be essential for spicule formation in previous studies [59,60]. Other members of these families have are more limited distribution and may be responsible for specific variations in matrix assembly and matrix properties. To the group of apparently indispensable proteins also belongs the carbonic anhydrase Sp-Cara7LA, several matrix metalloproteases, MSP130, and the glycine-rich protein(s) Sp-Hypp_2998/Hypp_2999 encoded in Glean3:18406/18407. Other proteins, such as phosphodontin [27] or a group of proteins with alternating Ala- and Pro-rich and acidic Gly-rich motifs described in tooth matrix [26] may be specific for a certain type of mineralized structure, in this case the sea urchin tooth. Of course minor components and components acting at the periphery of mineralization events, especially those with a catalytic activity, can also be important. This may include kinases, notch-like proteins and notch ligand-like proteins, members of the RAS family, ion channels, or phospholipases. The current proteomic inventories may be taken as a suggestion to determine the role of interesting proteins by suitable methods, such as the use of specific antibodies, inhibitors, or iRNAs. Finally, the more than 400 identified proteins in skeletal elements validate many previously predicted hypothetical proteins and confirm their existence.

## Abbreviations

CTLLD: C-type lectin-like domain; FDR: false discovery rate; MMP: matrix metalloproteases; PEP: posterior error probability; PM: primary mesenchyme; PMC: primary mesenchyme cells; SM: spicule matrix.

## Competing interests

The authors declare that they have no competing interests.

## Authors' contributions

KM conceived the study, performed organic matrix and peptide isolation and data acquisition. AJP and FHW prepared spicules. KM and AJP did database searches and annotations. All authors took part in the design of the study and were critically involved in data interpretation and manuscript drafting. All authors read and approved the final manuscript.

## Supplementary Material

Additional file 1 **Spicule matrix proteins identified with high confidence**. Click here for file

Additional file 2 **Tentatively identified spicule matrix proteins**. Click here for file

Additional file 3 **Protein data (xlsx-file)**. This file shows the MaxQuant-derived protein data such as protein scores, number of peptides, and molecular weight. Commonly occurring contaminants, such as human keratins or trypsin, were removed. From left to right the columns show Glean3 entry number (A), protein group number (B; groups may contain several Glean3 entries, representing closely related proteins that share peptides), peptide IDs (C; allow to locate a given peptide in Additional file 4:MaxQuant peptide data), sum of peptides given in the next columns (D; numbers separated by semicolon represent peptide numbers for different Glean3 entries in a group shown in column A and appear in the same order), sum of unique peptides and razor peptide (i.e. peptides shared by different proteins of a group; E), unique peptides (F), number of proteins in the respective group (G), numbers of peptides, razor peptides and unique peptides with different sequences or modifications (H-M), sequence coverage in % for calculated with all peptides, unique plus razor peptides and unique peptides alone (N-P), molecular weight of the identified protein (Q), length of the protein sequence for the leading protein (R), PEP score for the protein (S; a PEP of 0 is >4.9E-324), and intensity (sum of all identified peptide peaks; T). Cross-reference to Additional file 4 is provided by protein Group IDs and Peptide IDs. Click here for file

Additional file 4 **Peptide data**. This Excel sheet contains unique and razor peptide data. Peptides of common contaminants, such as human keratins or trypsin, were removed. A triple helical hydroxyproline-containing peptide belonging to Glean3:15708 and identified in a MaxQuant run including hydroxylation of proline as a variable modification is included with peptide ID Hyp1 at the end of this sheet. Cross-reference to Additional file 3 is provided by identical Peptide IDs (A) and Protein Group IDs (B) in both Excel sheets. Other columns show peptide sequence (C), peptide length (D), number of missed cleavages (E), peptide mass (F), Glean3 accession numbers (G), Glean3 accession number of the leading protein (if several related proteins of a group share peptides; H), uniqueness of the peptide (I), PEP score (J), Mascot score (K), distribution of a peptide over gel slices (L-AB, and intensity (sum of intensities of repeatedly sequenced peptides of the same sequence (AC). Click here for file

Additional file 5 **Compilation of proteins identified in *S. purpuratus *skeletal elements**. The proteins were compiled from proteomic analyses of spicule matrix (this report) and published data of spine, test and tooth proteomes [25-27]. ++, proteins among the 33% most abundant; +, present at lower abundance; (+), tentatively identified; -, not found. P, at least partially phosphorylated. Extracellular proteins and membrane proteins identified as major components in all matrices and therefore possibly of general importance for sea urchin mineralization events are framed in green. Major extracellular or membrane proteins specific for a single matrix and potentially interesting for assembly or mineralization are framed yellow. Minor proteins are not considered, because their presence or absence may also be caused by changes in instrumentation and evaluation software. The matrix of intact teeth is not included into these considerations because it may contain a higher proportion of contaminants from cellular debris [26]. Click here for file

Additional file 6 **Alignment of CTLLD sequences of matrix proteins**. This file shows a ClustalW2 alignment of the sequences of predicted CTLLD enlisted in Table 1 (A) and a tree representation for graphical visualization (B). Click here for file

Additional file 7 **Selected MS/MS spectra of SM30-D peptides**. This file contains a ClustalW2 alignment of SM30-D sequence variants and MaxQuant-annotated MS/MS spectra of unique SM30-D peptides. Click here for file

Additional file 8 **Alignment of Sp-Mmp18/19-like sequences**. Click here for file

Additional file 9 **Comparison of thombospondins Sp-Thsd7b and Sp-Thsd7b_2**. Click here for file
