# Supplementary material for: Proteomic analysis of sea urchin (Strongylocentrotus purpuratus) spicule matrix
Source: Proteome Sci. 2010 Jun 17;8:33. doi: 10.1186/1477-5956-8-33 (PMC2909932; doi:10.1186/1477-5956-8-33)
Supplement: Additional file 1 — Spicule matrix proteins identified with high confidence. [file 1477-5956-8-33-S1.DOCX]

**Spicule organic matrix proteins.**

| **Glean3_ entry** | **Protein** | **Presumed**  **compart-**  **ment ^2^** | **Unique pep-tides** | **Sequen-ce cover-age** | **Gel section ^3^** | **Protein**  **Score**  **(PEP)** | **emPAI** |
| --- | --- | --- | --- | --- | --- | --- | --- |
|  |  |  |  |  |  |  |  |
| 18811 **^1a,b,c,d^** | Sp-Sm50 | extracellular | **13** | 69% | 1-13 | <4.9E-324 | >1.6E6 |
| 13821 **^1a,bc,d^** | Sp-Msp130_1; shares 14 peptides with Glean3:02088 (Sp-Msp130); GPI | extracellular | **30** | 37% | 4,5,8,  11-14 | <4.9E-324 | 22387.2 |
| 00434 | Hypothetical protein, acidic (pI 4.4) | unknown | **2** | 31% | 1-9 | 1.4E-171 | 9999.0 |
| 05990 **^1a,b,c,d^** | Sp_Sm29/SM29 | extracellular | **11** | 41% | 9-15 | <4.9E-324 | 4216.0 |
| 18406 **^1a,b,c,d^** | Sp-Hypp_2998; 30% Gly; shares peptides with 18407 (Sp-Hypp_2999) | transmembrane | **19** | 69% | 10-13 | <4.9E-324 | 2609.2 |
| 13822 **^1a,b,c,d^** | Sp-Msp130r1; GPI | extracellular | **18** | 36% | 4-7,9,  11,12 | <4.9E-324 | 2153.4 |
| 13823 **^1a,b,c,d^** | Sp-Msp130r3; shares peptides with Glean3:06387 (Sp-Msp130L/MSP130-related-3); GPI | extracellular | **43 ^4^** | 54% **^4^** | 3-15 | <4.9E-324 | 1192.8 **^4^** |
| 12518 **^1a,b,c,d^** | Sp-Cara7LA (carbonic anhydrase) | extracellular | **14** | 32% | 10-14 | <4.9E-324 | 999.0 |
| 13825 **^1a,b,c,d^** | Sp-Clect_76, domain: C-lectin; pI 9.9; 12% Gly, 16% Pro | extracellular | **7** | 29% | 6-15 | 4.5E-201 | 718.7 |
| 18810 **^1a,b,c,d^** | Sp-Sm32 | extracellular | **3** | 13% | 3-14 | <4.9E-324 | 680.3 |
| 06387 **^1a,b,c,d^** | Sp-Msp130L (primary mesenchyme specific protein 130-like/MSP130-related-3); shares peptides with Glean3:13823 (Sp-Msp130r3); GPI | extracellular | **44 ^4^** | 55% **^4^** | 4-13 | <4.9E-324 | 582.4 **^4^** |
| 16506 **^1a,b,c,d^** | Sp-Msp130r2; shares 15 peptides with Glean3:21385 (SpApl; all 15 peptides from N-terminal half!); GPI | extracellular | **24 ^4^** | 60% **^4^** | 5-14 | <4.9E-324 | 425.2 |
| 30147 | Sp-Pm27; PM27 | extracellular | **7** | 39% | 1-3,6,7,  9-13 | 4.4E-109 | 315.2 |
| 21385 **^1a,b,c,d^** | Sp-ApL/MSP130-related-2, shares 15 peptides with Glean3:16506 (Sp-Msp130r2); GPI | extracellular | **22 ^4^** | 66% **^4^** | 4-6,9-14 | <4.9E-324 | 208.6 **^4^** |
| 09549 **^1a,(c),d^** | Similar to Sdcbp-prov (syndecan-binding protein, syntenin); domains: PDZ_signaling; N-term:N-AcSer2 | membrane-  associated | **11** | 38% | 10-13 | 8.2E-302 | 99.0 |
| 18813 **^1a,b,c,d^** | Sp-Sm37 | extracellular | **10** | 50% | 3,5-15 | <4.9E-324 | 83.8 |
| 05989 **^1a,b,c,d^** | Sp-Clect_13/similar to SM29 | extracellular | **5** | 30% | 11-15 | <4.9E-324 | 80.1 |
| 05577 | Sp-Mmp18/19L3/similar to matrix metalloprotease 14; shares 2 peptides with Glean3:09924 (Sp-Mmp18/19L5) and 3 peptides with Glean3:09925 (Sp-Mmp18/19L4) | extracellular | **7 ^4^** | 26% **^4^** | 8-14 | 1.7E-212 | 76.4 **^4^** |
| 13669 **^1a,b,c,d^** | Sp-Mt1-4/MmpL5 (matrix metalloproteases); shares 1 peptide with Glean3:13670 (Sp-Mt1-4/MmpL6) | extracellular/  transmembrane | **12 ^4^** | 21% **^4^** | 9-15 | 1.3E-297 | 74.0 **^4^** |
| 11163 **^1(a),(b),c,d^** | Sp-Clect_25/hypothetical protein; domain C_lectin | extracellular | **3** | 32% | 3,6,9,  11-14 | 4.6E-197 | 67.1 |
| 28749 **^1a,b,c,d^** | Sp-Mt5/MmpL2/similar to matrix metalloproteases 14 | extracellular/  transmembrane | **17** | 31% | 8-15 | <4.9E-324 | 66.0 |
| 25068 **^1a,b,c^** | Sp-Ttrspn_19 (tetraspanin; emPAI and coverage calculated for the extracellular part) | membrane | **3** | 22% | 1-15 | 2.6E-29 | 55.2 |
| 05538 **^1a,b,c,d^** | Sp-Lrr/Igr_2 | transmembrane | **21** | 38% | 4-15 | <4.9E-324 | 52.8 |
| 13670 **^1a,b,c,d^** | Sp-Mt1-4/MmpL6 (matrix metalloprotease); shares 1 peptide with Glean3:13669 (Sp-Mt1-4/MmpL5) | extracellular/  transmembrane | **17 ^4^** | 36% **^4^** | 7-15 | <4.9E-324 | 50.1 **^4^** |
| 14160 | Hypothetical protein; domain: partial annexin (A7) | membrane-associated | **3** | 40% | 1,6,7,  9-11,13 | 8.4E-150 | 45.4 |
| 04867 **^1a,b,c,d^** | Sp-Sm30E (SM30-E) | extracellular | **8** | 25% | 7,8,10,11 | 2.1E-284 | 37.3 |
| 07682 **^1a,b,c,d^** | Sp-CbpdEL/hypothetical protein, domain: carboxypeptidase (peptidase_M14) | extracellular | **12** | 35% | 7-15 | 1.2E-304 | 36.9 |
| 28748 **^1a,b,c,d^** | Sp-Mt1-4/MmpL7/matrix metalloprotease 16 | extracellular | **22** | 40% | 7-15 | <4.9E-324 | 36.3 |
| 05991 **^1a,b,(c),d^** | Sp-Clect_14/similar to SM29 | extracellular | **6** | 41% | 11-15 | 7.9E-107 | 26.8 |
| 04746 **^1a,b,c^** | Sp-Fn3/Igf_29/similar to *P. lividus* FGF receptor 2 | extracellular/  transmembrane | **13** | 38% | 8-14 | <4.9E-324 | 26.2 |
| 22047 **^1(b),c,(d)^** | Sp-Pla2g1b_8/similar to phospholipase A2 | extracellular/  membrane-ass. | **4** | 20% | 1-14 | 1.9E-202 | 20.5 |
| 25927 **^1c^** | Hypothetical protein/similar to CG18405-PB; domain: sema (C-term); same protein as Glean3:25926? | extracellular/  transmembrane | **10** | 42% | 1,3-8,11 | <4.9E-324 | 18.3 |
| 20457 **^1a,b,c^** | Sp-B7L3/hypothetical protein; domains: IG | transmembrane | **10** | 23% | 4-11 | <4.9E-324 | 16.0 |
| 07484 **^1a,b^** | Sp-Cycpln/cyclophilin 1 | extracellular (ER) | **4** | 28% | 13-15 | 1.1E-235 | 15.7 |
| 21496 **^1a,b,c,d^** | Sp-Polyq/ubiquitin; shares petide set with Glean3:04721 (Sp-Rps27a/ribosomal protein S27a), Glean3:18079 (Sp-Drps27a) and Glean3:15276 (Sp-Ubq/RL40fp) | intracellular | **4** | 62% | 1-16 | 3.1E-117 | 13.7 |
| 09925 | Sp-Mmp18/19L4 (similar to MMP-14); shares 1 peptide with Glean3:05723 (Sp-Mmp18/19L6), 3 peptides with Glean3:09924 (Sp-Mmp18/19L5) and 3 peptides with Glean3:05577 (SpMmp18719L3) | extracellular | **8 ^4^** | 20% **^4^** | 8-11 | <4.9E-324 | 13.4 **^4^** |
| 12144 **^1b,c,d^** | Sp-EhH4_33/histone H4 family; peptide set shared with many entries (see Additional file 3) | intracellular | **5** | 37% | 1,2,6-8,  13,15,16 | 9.3E-160 | 12.9 |
| 25966 **^1a,b,c,d^** | Sp-Lrr15L (Leucine-rich repeat-containing protein 15_like) | transmembrane | **23** | 25% | 1,3-6,  11-15 | <4.9E-324 | 12.9 |
| 01892 **^1a,b,c^** | Sp-Sema6d (semaphorin 6D) | extracellular/  transmembrane | **9** | 29% | 3-9,13,14 | 9.5E-235 | 12.6 |
| 25454 | Sp-Thsd7b (Thrombospondin type-1 domain-containing protein 7B) | extracellular/  transmembrane | **7** | 28% | 1-9 | <4.9E-324 | 11.9 |
| 25926 **^1(a),c^** | Hypothetical protein/similar to CG18405-PB, domain: sema (N-term); same protein as Glean3:25927? | extracellular/  transmembrane | **7** | 50% | 1-9,13 | 2.0E-273 | 11.9 |
| 17641 | Sp_Hypp_887 | extracellular | **15** | 33% | 3-5,7-10,  13,14 | <4.9E-324 | 11.7 |
| 02788 **^1b,c^** | Sp-Btub1 (β-tubulin); shares 12 peptides with Glean3:00062 (Sp-Btub3) and 6 peptides with Glean3:14285 (Sp-Tubb2a; tubulin C) | intracellular | **12 ^4^** | 34% **^4^** | 1,2,8,10,14 | 3.4E-187 | 10.4 **^4^** |
| 10203 | Sp-Yp30l/similar to HLC-32/yolk granule protein YP30 | extracellular | **13** | 45% | 10-15 | 1.6E-302 | 10.2 |
| 00438 **^1a,b,c^** | Sp-Hypp_302/similar to peptidylaminoacyl-L/D isomerase | transmembrane  (ER) | **7** | 19% | 7-10,13 | 7.5E-236 | 9.0 |
| 04885 | Sp-Apod/similar to apolipoprotein D | extracellular | **4** | 16% | 7,8 | 6.9E-38 | 9.0 |
| 07441 | Similar to MGC139263 protein; domain: annexin (partial?) | membrane-associated | **3** | 33% | 1,4-7,  9-11,13 | 1.8E-143 | 9.0 |
| 23052 **^1a,b,c,d^** | Hypothetical protein | transmembrane | **4** | 32% | 1,11,  13-15 | 4.1E-38 | 9.0 |
| 13716 **^1(a)^** | Sp-Sorc/similar to sorcin | intracellular/  membrane-associated (ER) | **6** | 35% | 12,13 | 4.1E-63 | 9.0 |
| 24991 **^1c^** | Hypothetical protein; N-term:N-Ac-Met_1_; phospho-Ser_144_ | extracellular/  transmembrane | **4** | 29% | 1-5,  11-16 | 4.3E-40 | 9.0 |
| 05228 **^1a,b,c^** | Sp-CalsynteninL1/hypothetical protein; domains: LamG, cadherin; shares 1 peptide with Glean3:23005 (Sp-calsynteninL2) | transmembrane/  extracellular | **8** | 27% | 3-8,12 | 7.5E-241 | 7.9 |
| 25962 **^1a,b^** | Sp-Anxa7_2 (annexin)/similar to MGC139263 protein; shares 3 peptides with Glean3:00475 | membrane-associated | **10** | 22% | 1,5-7,  9,10 | 4.9E-140 | 7.5 |
| 19739 | Sp-Thsd7b (thrombospondin)/similar to RIKEN cDNA D130067I03 gene, partial; shares 2 peptides with Glean3:25454 (Sp-Thsd7b_2) | extracellular | **7** | 15% | 1-10 | 3.7E-278 | 7.4 |
| 07355 | Sp-Unk_38; domain: ZnMc-adamalysin_II_like | extracellular | **4** | 16% | 1-3,  5-10,15 | 3.6E-151 | 6.7 |
| 17630 | Sp-Hypp_885 | transmembrane | **10** | 17% | 3-9,  13-15 | <4.9E-324 | 6.5 |
| 20701 **^1(a)^** | Hypothetical protein | unknown | **4** | 21% | 4-6,9,13 | 1.4E-59 | 6.5 |
| 06123 | Sp-AchE (acetylcholine esterase)/similar to cholinesterase 2 | extracellular | **11** | 27% | 5-7,9,13 | <4.9E-324 | 6.0 |
| 00164 **^1a,(b),(d)^** | Sp-Clect/similar to SM30 | extracellular | **2** | 10% | 1,3-9,  12,13 | 9.6E-40 | 5.8 |
| 09516 | Sp-CopineA/hypothetical protein; domains: C2,vWFA | membrane-associated | **14** | 36% | 6-8 | <4.9E-324 | 5.1 |
| 26140 | Sp-Hypp_2821; domain: SBP_bac_3 (bacterial periplasmic transporter) | extracellular/  transmembrane | **5** | 23% | 10-14 | 5.6E-138 | 5.1 |
| 19655 **^1a^** | Sp-AnL18 (astacin-like) | extracellular | **8** | 48% | 4-7,  9-15 | 1.1E-59 | 4.4 |
| 00439 **^1a,b,c,(d)^** | Sp-FcgbpL (Fc fragment of IgG-binding protein-like) shares 2 peptides with Glean3:04158 and 1 peptide with Glean3:04159 | transmembrane | **8** | 21% | 5-10,14 | 7.6E-189 | 4.3 |
| 09924 | Sp-Mmp18/19L5 (similar to MMP14), shares 2 peptides with Glean3:05577 (Sp-Mmp18/19L3),1 peptide with Glean3:05723 (Sp-Mmp18/19L6) and 3 peptides with Glean3:09925 (Sp-Mmp18/19L4) | extracellular | **5 ^4^** | 14% **^4^** | 8-11 | 3.8E-27 | 4.3 **^4^** |
| 03914 | Sp-Plscr1 (phospholipid scramblase 1)/hypothetical protein; shares 2 peptides with Glean3:05017 (Sp-Plscr1_1) | transmembrane | **3** | 24% | 3,4,9-13 | 5.7E-111 | 4.0 |
| 26982 | Sp-SerinC1; potential transmembrane regions excluded from calculations; peptides from extracellular loops | membrane (ER) | **2** | 15% | 1,3-9 | 1.0E-86 | 3.6 |
| 06103 **^1a,b^** | Hypothetical protein/similar to calsynthenin-1 (aa113-217) | transmembrane | **5** | 22% | 3-6,  11-16 | 1.7E-30 | 3.6 |
| 19987 | Hypothetical protein | unknown | **5** | 24% | 1-5,  10-13 | 1.6E-57 | 3.6 |
| 26365 | Sp-Sri (sorcin); domains: EFh; shares peptides with Glean3:26366 | intracellular/  membrane (ER) | **3** | 13% | 13 | 3.8E-59 | 3.6 |
| 25235 **^1a,b,c,d^** | Sp-Npnt (nephronectin)/similar to Egfl6-prov protein | extracellular | **14** | 20% | 1,3,4,  8-12 | 2.3E-279 | 3.5 |
| 02783 | Sp-NotchL_1 (notch-like 1)/similar to fibropellin Ib; domains: MAM, EGF | transmembrane | **10** | 11% | 1,3-7,  11-13 | 4.2E-161 | 3.3 |
| 27169 **^1a,b^** | Sp-Hmcn1L_4 (hemicentin-like)/similar to fibulin-6 (aa1-358); domain: IGcam | transmembrane | **7** | 26% | 8,9,  11-14 | 1.0E-204 | 3.2 |
| 25246 | Sp-Lman2/similar to vesicular mannose-binding lectin | transmembrane  (ER,Golgi) | **5** | 17% | 10,11,  13-15 | 1.6E-25 | 3.1 |
| 11106 **^1(a),b,d^** | Sp-Anxn (annexin)/similar to annexin A4; shares 2 petides with Glean3:11107 (Sp-Anxn_1) | membrane-associated | **12** | 48% | 10,11 | <4.9E-324 | 3.1 |
| 00049 | Sp_TrypL (trypsin-like serine protease with CCP repeats)/similar to complement component 2, factor B | extracellular | **10** | 22% | 9-14 | 5.6E-44 | 3.0 |
| 03402 | Sp-Sdc (syndecan) | transmembrane | **2** | 16% | 10,11 | 2.2E-117 | 3.0 |
| 26000 **^1a,b,c,d^** | Sp-Hypp_1164; domain:IG | transmembrane | **8** | 18% | 9-14 | <4.9E-324 | 2.8 |
| 00826  00827 | Sp-Sm30B (SM30-α)  Sp-Sm30C (SM30-β); sequences are 97% identical | extracellular | **2** | 11%% | 6,9-14 | 4.3E-36 | 2.7 |
| 27906 **^1a,b,c,d^** | Sp-C-lectin/PMC1 | extracellular | **4** | 13% | 1-15 | 3.2E-56 | 2.6 |
| 00992 | Sp-AnthrtrL_1/similar to anthrax toxin receptor 1;  the entry contains the extracellular domain | extracellular/  transmembrane | **3** | 16% | 8,9 | 9.3E-139 | 2.6 |
| 06370 | Sp-Ncam/similar to Ncam-140 | transmembrane | **9** | 14% | 4-9,  11-13 | 5.7E-171 | 2.6 |
| 07451 | Sp-Hypp_1723; domains:Cys-rich secretory protein, EGF | transmembrane/  extracellular | **3** | 13% | 1,3-9,16 | 1.4E-90 | 2.6 |
| 08857 | Sp-Timp3b (tissue inhibitor of metalloproteases) | extracellular | **4** | 18% | 13 | 8.0E-15 | 2.6 |
| 15321 **^1a^** | Sp-Celsr1 | transmembrane | **10** | 14% | 9-11,13,  15 | 6.9E-90 | 2.6 |
| 21041 | Hypothetical protein; domain: annexin; similarity to A11 | membrane-associated | **2** | 22% | 5-7,9-11 | 3.2E-40 | 2.6 |
| 24653 | Sp-C1qL_4 (complement C1q-like) | extracellular | **6** | 20% | 9,11,12 | 1.8E-91 | 2.6 |
| 24565 **^1a,b,c,d^** | Sp-Cd109L/similar to thioester-containing protein; domain: A2M_2 (α-macroglobulin) | membrane/  extracellular | **8** | 13% | 3-14 | 1.8E-219 | 2.6 |
| 03504 | Similar to Jedi protein; shares 1 peptide with Glean3:08666 (Sp-Pearl1L, platelet endothelial aggregation receptor 1-like) | transmembrane | **2** | 9% | 4-11 | 1.8E-52 | 2.5 |
| 12138 | Sp-LamG/Egff2/similar to DEAH box polypeptide 33; shares 1 peptide with Glean3:15404 /Sp-LamG/Egff3) | extracellular/  transmembrane | **4 ^4^** | 8% **^4^** | 3-5 | 6.0E-118 | 2.5 **^4^** |
| 00469 **^1a,b,c^** | Sp-Igcamr/similar to novel hemicentin/similar to cell adhesion molecule OCAM | extracellular/  transmembrane | **5** | 12% | 4,11-16 | 6.7E-73 | 2.2 |
| 22779 | Sp-Pdcd6 (programmed cell death 6)/similar to apoptosis-linked gene 2 | intracellular/  membrane-ass. | **4** | 22% | 13,14 | 2.0E-28 | 2.2 |
| 25574 | Sp-Rps14 (ribosomal protein S14) | intracellular | **2** | 16% | 1,2,10,14 | 6.1E-13 | 2.2 |
| 23016 **^1a,b,d^** | Sp-Frem1L/88% sequence identity to *L. variegatus* ECM3/FREM2 | extracellular/  transmembrane | **20** | 16% | 3-6,  11-14 | <4.9E-324 | 2.1 |
| 05238 **^1a,c^** | Sp-Plxdc2 (plexin domain-containing 2) | transmembrane | **3** | 10% | 1-14 | 1.2E-282 | 1.9 |
| 16397 | Sp-Hypp_2259/similar to pleiotrophin | extracellular | **3** | 12% | 1,3-7,9 | 1.1E-18 | 1.9 |
| 12486 **^1a,b^** | Sp-Hypp_1035/similar to MEGF11 protein; domain: EMI (Cys-rich domain of EMILINS) | extracellular | **3** | 13% | 12,13 | 2.2E-25 | 1.8 |
| 24181 **^1(a)^** | Sp-Nell1L/similar to protein kinase c-binding protein nell1; domains: EGF, LamG, VWC | extracellular/  transmembrane | **7** | 15% | 1,3-7 | 1.4E-81 | 1.8 |
| 26001 | Hypothetical protein; domains: annexin | membrane-associated | **3** | 22% | 6,7,10,  11 | 3.4E-12 | 1.8 |
| 28887 **^1a,(b)^** | Hypothetical protein/similar to cytoplasmic cystatin | intracellular | **2** | 25% | 14,15 | 2.1E-78 | 1.7 |
| 13857 | Sp-Vps28/similar to vacuolar protein sorting 28 | intracellular/  membrane-ass. | **5** | 31% | 12,13 | 5.7E-22 | 1.6 |
| 26072 **^1a^** | Sp-EceL(endothelin converting enzyme)/similar to neprilysin; domain: Peptidase_M13 | transmembrane/extracellular | **11** | 18% | 3-6 | <4.9E-324 | 1.6 |
| 20636 | Sp-CopineD/similar to Copine VIII; shares 2 peptides with Glean3:08019 (Sp-CopineL) | intracellular/  membrane-ass. | **9 ^4^** | 18% **^4^** | 1,3-7 | 2.8E-147 | 1.6 **^4^** |
| 01093  11573 | Hypothetical protein; domains: MAM (meprin,A5 protein, and tyrosine phosphatase Mu); very acidic Glu-rich motif in C-terminus (aa191-220) | unknown | **2** | 11% | 12,13 | 7.2E-26 | 1.5 |
| 01796 **^1a,b,(c),d^** | Sp-Frem2 (Fras1-related extracellular matrix protein 2) | transmembrane/extracellular | **15** | 22% | 3-6,11 | 6.4E-221 | 1.5 |
| 00884 | Sp-Ttrspn (tetraspanin); emPAI and coverage calculated for extracellular domain | membrane | **2** | 32% | 3,4,10,  11 | 4.6E-14 | 1.5 |
| 25915 | Sp-Kiaa0174L; domain: DUF292; 15% Pro; N-term: Acetyl-Met1 | intracellular/  membrane | **4** | 12% | 8,9,13 | 2.3E-22 | 1.5 |
| 26984 **^1(a)^** | Sp-QpctlL (glutaminyl-peptide cyclotransferase-like); shares 1 peptide with Glean3:23956 (Sp-Hypp_208) | transmembrane | **4** | 15% | 10-15 | 3.2E-84 | 1.5 |
| 07550 | Sp-Pef1_1 (peflin)/similar to apoptosis-linked gene 2 | intracellular/  membrane-ass. | **3** | 11% | 10-13 | <4.9E-324 | 1.4 |
| 08505 **^1a,b^** | Sp-Cys-rich_fgfr (similar to Golgi apparatus protein 1)/ similar to ENSANGP00000020376, partial; shares 4 peptides with Glean3:18130 (Sp-Cys-rich_fgfr_2) and 1 peptide with Glean3:19782 (Sp-Cys-rich_fgfr_3) | transmembrane  (Golgi) | **12 ^4^** | 20% **^4^** | 3-15 | 4.0E-232 | 1.4 **^4^** |
| 00619 | Sp-ApdpL/similar to aminopeptidase P; GPI? | extracellular | **2** | 13% | 5,8,11-13 | 5.5E-70 | 1.3 |
| 23466 | Sp-calu (calumenin)/similar to cardiac calumenin; shares 1 peptide with Glean3:18461 | extracellular  (ER lumen) | **3** | 15% | 9-11,13,  14 | 1.9E-15 | 1.3 |
| 13077 **^1a,b^** | Sp-Hypp_87/similar to TFP250/thrombospondin; domains: CCP, EGF; shares 1 peptide with Glean3:16774 (Sp-Hypp3379) | extracellular | **9 ^4^** | 8% **^4^** | 1-6,  11-14 | 9.4E-162 | 1.3 **^4^** |
| 07341 | Sp-Caspr/similar to neurexin IV, shares peptide set with Glean3:04145 | transmembrane | **4** | 7% | 3,4 | 1.4E-15 | 1.2 |
| 06172 **^1(a),b^** | Sp-Cfln/similar to cofilin-related; domain: actin depolymerization factor | intracellular | **3** | 24% | 13,14 | 3.8E-34 | 1.2 |
| 06145 | Sp-Kiaa1324L/hypothetical protein; domains: mannose 6-phosphate receptor, growth factor receptor, TNF receptor-like | transmembrane | **8** | 10% | 1,3-6 | 3.1E-138 | 1.2 |
| 05779 | Sp-Rap1/similar to Rap1b-prov | intracellular | **3** | 18% | 13,16 | 1.2E-18 | 1.2 |
| 13819 **^1c^** | Sp-Eno1 (α-enolase); shares 1peptide with Glean3:13820 (Sp-Eno3, β-enolase) | intracellular | **2** | 10% | 1-3,6,8-10,14,16 | 2.5E-58 | 1.2 |
| 16790 | Sp-Rac; shares 1 peptide with Glean3:10545 (Sp-Racl1) | intracellular | **2** | 9% | 10,13 | 7.1E-12 | 1.2 |
| 17072 | Sp-Rab8; shares peptides with 7 other Rab proteins (see Additional file 3) | intracellular | **4 ^4^** | 24% **^4^** | 8,10,13,  14 | 1.5E-29 | 1.2 **^4^** |
| 09477 | Sp-Hsp701C; shares peptides with many related entries (see Additional file 3) | intracellular | **5 ^4^** | 9% **^4^** | 1-16 | 5.7E-187 | 1.2 **^4^** |
| 15404 **^1a,b^** | Sp-LamG/Egff3/similar to DEAH box protein 33 | transmembrane | **11** | 13% | 1,3-7,9,  13 | 1.2E-203 | 1.1 |
| 22576 | Hypothetical protein; domains: Sod_Cu, DEATH | extracellular | **8** | 11% | 3-6 | 8.6E-116 | 1.1 |
| 05420 **^1a,b^** | Sp-Srcr42/similar to scavenger receptor cysteine-rich protein type 12 | transmembrane | **6** | 12% | 1-5 | 1.1E-67 | 1.0 |
| 11963 | Sp-Tsg101 (tumor susceptibility gene 101); domains: UBCc (ubiquitin-conjugating E2) | intracellular | **5** | 13% | 8,13 | 6.2E-74 | 1.0 |
| 13474 | Hypothetical protein; domains: low density lipoprotein receptor | extracellular | **2** | 17% | 10 | 7.8E-23 | 1.0 |
| 14869 **^1a,b,c^** | Sp-prxdxn (thioredoxin-peroxidase); shares 1 peptide with Glean3:06211 (Sp-Pxr4) | unknown | **2** | 10% | 1-16 | 9.4E-69 | 1.0 |
| 15708 | Sp-Col4a2 (alpha2(IV)-like collagen) | extracellular | **3** | 11% | 4,9-11 | 1.2E-23 | 1.0 |
| 20140 | Sp-pdcd6ip (programmed cell death 6 interacting); shares 1 peptide with Glean3:09210 (Sp-Pdcd6ip_2) | intracellular/  membrane | **7** | 9% | 4-6,  10,11,13 | 3.7E-222 | 1.0 |
| 23115 **^1a,b,c,d^** | Sp-Ptpriz (protein tyrosine phosphatase, receptor type)/ similar to brain RPTPmam4 isoform II | transmembrane | **12** | 7% | 4-13 | <4.9E-324 | 1.0 |
| 23217 **^1(b),c^** | Sp-Ran | intracellular | **3** | 14% | 10,14 | 1.4E-10 | 1.0 |
| 01312 | Sp-ClvhH2b; shares peptides with many related entries  (see Additional file 3) | intracellular | **2** | 16% | 1,6,15 | 2.7E-05 | 0.9 |
| 03084 **^1(a),(b)^** | Sp-Gpc1246_1/glypican-6; GPI? | extracellular | **5** | 12% | 4,10,11,  13,15 | 6.2E-87 | 0.9 |
| 03555 | Hypothetical protein/similar to SH2/SH3 adapter Grb3; domains: SH2,SH3 | intracellular | **2** | 16% | 11,13 | 8.4E-38 | 0.9 |
| 06812 **^1a,b,c^** | Sp-Kirrel2L (kin of IRRE-like 2-like) | transmembrane | **8** | 10% | 4,8-15 | 2.7E-174 | 0.9 |
| 11147  15227 | Hypothetical protein | extracellular/  transmembrane | **2** | 11% | 4-8 | 1.4E-10 | 0.9 |
| 14602 **^1a^** | Sp-Srcr115 (scavenger receptor Cys-rich) | extracellular/  transmembrane | **4** | 9% | 1-5 | 1.7E-62 | 0.9 |
| 18964 **^1a,b^** | Sp-Fk506bp2/similar to MGC53657 | extracellular/  membrane (ER) | **2** | 21% | 14 | 1.2E-05 | 0.9 |
| 24995 | Sp-KirrelL (kin of IRRE_like); domains: IG | transmembrane | **2** | 6% | 8,11,12 | 2.6E-60 | 0.9 |
| 08101 | Sp-Fam78a (similar to Family with sequence similarity 78, member A) | extracellular | **2** | 6% | 11 | 4.1E-18 | 0.8 |
| 19319 | Sp-Hypp_2431;domain: partial neurexophilin, IG | unknown | **4** | 12% | 3-6  10-12 | 9.0E-16 | 0.8 |
| 00752 | Sp-Hypp_313 (hypothetical protein 313); 12% Ser | transmembrane | **3** | 6% | 4,6-9 | 1.2E-189 | 0.8 |
| 23288 | Sp-Hypp_2210; shares 1 peptide with Glean3:21860; domains:CCP/Sushi | transmembrane | **2** | 9% | 11,12 | 4.0E-22 | 0.8 |
| 24042 | Similar to heparan sulfate D-glucosaminyl 3-O-sulfotransferase-2 | transmembrane  (Golgi) | **4** | 16% | 12-14 | 4.2E-26 | 0.8 |
| 05723 | Sp-Mmp18/19L6; shares 1 peptide with Glean3:09924 (Sp-Mmp18/19L5) and Glean3:09925 (Sp-Mmp18/19L4) | extracellular | **3 ^4^** | 9% **^4^** | 9 | 7.9E-13 | 0.8 **^4^** |
| 00310 | Sp-pdgfr/vegfrL/similar to Fit-1 tyrosine kinase and *P.* *lividus* VEGF receptor | transmembrane | **10** | 8% | 3-8,13 | 8.1E-267 | 0.7 |
| 04758 **^1(a)^** | Sp-Fn3f_9; domains: hyalin and fibronectin | transmembrane | **7** | 10% | 7,11-13 | 1.2E-37 | 0.7 |
| 05008 | Sp-Xpapd2Lmb-3/similar to aminopeptidase P | extracellular/  membrane-ass. | **4** | 15% | 5-10,13,  14 | 1.3E-22 | 0.7 |
| 04859 | Sp-Plekhb2 (pleckstrin homology domain-containing, family B, member 2) | extracellular  /membrane-  associated | **2** | 7% | 10,14 | 4.3E-05 | 0.7 |
| 05296 **^1a^** | Sp-Atp5b/ATP synthase β subunit | membrane  (mito) | **4** | 11% | 1,10,14 | 5.7E-23 | 0.7 |
| 19369 | Sp-Rndzvn (rendezvin); all peptides match to the C-terminus of this entry, which is not covered by SPU_019369; may not be part of rendezvin! | extracellular | **7** | 5% | 13,14 | 2.0E-77 | 0.7 |
| 22778 | Sp-Chmp4b (charged multivesicular body protein 4b) | intracellular/  membrane | **2** | 18% | 11,12 | 3.9E-12 | 0.7 |
| 28184 | Sp-SmocL (osteonectin/SPARC family) | extracellular | **3** | 7% | 7-9,11,  13,14 | 1.2E-111 | 0.7 |
| 00595 **^1b,c^** | Sp-EF1a (elongation factor 1A); shares 2 peptides with Glean3:09949 (Sp-Ef1a_1) | intracellular | **4** | 17%% | 1-11,13,  14,16 | 3.3E-15 | 0.6 |
| 00152 **^1(a)^** | Similar to notch; domains: EGF; shares 1 peptide with Glean3:13510 (Sp-NotchL_4) | transmembrane | **2** | 1.6% | 2,3,5 | 4.8E-70 | 0.6 |
| 06029 | Sp-Knkl (knickkopf-like); shares 1 peptide with Glean3:05664 (Sp-Hypp_1594) | transmembrane | **2** | 3% | 5,6,11,  12,14 | 5.8E-22 | 0.6 |
| 11256 **^1(b)^** | Similar to phospholipase A2 | extracellular/  membrane-ass. | **2** | 16% | 13,14 | 1.9E-08 | 0.6 |
| 11332 **^1a,b,c,d^** | Sp-Clca1_3 (Ca^2+^-activated chloride channel/regulator?) | transmembrane | **5** | 7% | 11-15 | 2.9E-95 | 0.6 |
| 12112 **^1b,d^** | Sp-Rab5l; shares 1 peptide with Glean3:27060 (Sp-Rab5) | intracellular | **2** | 10% | 3,8,10,13 | 9.2E-43 | 0.6 |
| 14594 **^1(a),b,c^** | Sp-14-3-3_1; peptide set partially overlapping with Glean3:03825 (Sp-14-3-3ε), Glean3:22537 (Sp-14-3-3_2) and Glean3:11631 | intracellular | **3 ^4^** | 10% **^4^** | 1-3,5-8,  10-16 | 1.7E-47 | 0.6 |
| 10062 | Sp-Srcr83/SRCR membrane form 2; shares 1 peptide with Glean3:16374 (Sp-Srcr134), Glean3:09354 (Sp-Scrc75), Glean3:16373 (Sp-srcr133) and Glean3:15153 | extracellular/  transmembrane | **4** | 8% | 1-10 | 4.4E-30 | 0.6 |
| 12356 | Sp-C1orf58/hypothetical protein; domain: BRO1 | unknown | **5** | 15% | 7,8,10,15 | 3.2E-14 | 0.6 |
| 13662 | Sp-Rps3/similar to ribosomal protein S3 | intracellular | **4** | 14% | 1,2,10,14 | 8.4E-135 | 0.6 |
| 15902 | Sp-Hypp_2238/similar to neurotrimin | transmembrane | **2** | 7% | 4,5 | 4.4E-29 | 0.6 |
| 18516 | Sp-Prss12L_2 (serine protease 12-like)/similar to SRCR domain protein, membrane form 2; domains:SRCR, WSC, CCP | transmembrane | **3** | 7% | 8,9 | 6.8E-104 | 0.6 |
| 22598 **^1a^** | Sp-FReD/similar to angiopoietin-like protein, partial | extracellular | **2** | 8% | 10,11 | 1.1E-41 | 0.6 |
| 23910 | Sp-Hypp_1098; domains: IG,IG_like | transmembrane | **2** | 6% | 5-8,10 | 6.0E-38 | 0.6 |
| 24564 | Sp-Cd109L_2/similar to thioester-containing protein; domain: A2M_N, A2M_N_2 (α-macroglobulin) | extracellular | **4** | 7% | 3-6 | 1.2E-16 | 0.6 |
| 27885 **^1a^** | Sp-Cp (ceruloplasmin)/hephaestin-like | extracellular/  transmembrane | **7** | 8% | 5,8 | 1.0E-24 | 0.6 |
| 09165 | Sp-Hsp701A; shares 1 peptide with Glean3:09164 (Sp-Hsp701E) and 2 peptides with Glean3:09477 (Sp-Hsp701C) | intracellular | **4 ^4^** | 7% **^4^** | 1,10,14 | 1.2E-74 | 0.6 **^4^** |
| 02550 | Sp-MacpfB.1/similar to apextrin; shares 2 peptides with Glean3:16546 (Sp-MacpfB.3/similar to apextrin) andGlean3:15144 (Sp-MacpfB.2/similar to apextrin) | extracellular/  transmembrane | **3** | 7% | 7,11 | 1.5E-66 | 0.5 |
| 17083 | Sp-Plscr3 (scramblase); shares 1 peptide with Glean3:09119 (Sp-Pppl_252; pol polyprotein-like_252) and Glean3:24989 (Sp-Rdh8_9; retinol dehydrogenase) | membrane | **2** | 10% | 11-13 | 1.2E-21 | 0.5 |
| 03918 **^1a,b,(c),d^** | Sp-Lrp8 (low density lipoprotein receptor-related 8/similar to vitellogenin receptor) | transmembrane | **2** | 8% | 1,3-9 | 9.9E-67 | 0.5 |
| 06030 | Sp-Megf6-2 (Multiple epidermal growth factor-like domains 6) | extracellular | **7** | 7% | 4,5 | 4.1E-38 | 0.5 |
| 20978 | Sp-LrpL_3/similar to alpha-2-macroglobulin receptor; shares 2 peptides with Glean3:13664 | transmembrane | **22** | 6% | 1-7 | 2.9E-279 | 0.5 |
| 24022 | Sp-Hypp_1101/similar to nephrin; domains: IGcam | transmembrane | **5** | 5% | 3,15 | 6.2E-47 | 0.5 |
| 25499 | Sp-Hypp_1150; domains: DM13, DOMON, partial SRCR | extracellular | **3** | 5% | 4 | 5.4E-17 | 0.5 |
| 26944 **^1d^** | Sp-Arf1l1 (ADP-ribosylation factor 1_like 1); shares 1 peptide with Glean3:00552 (Sp-Arf1), Glean3:03162 (Sp-Arf1l2) and Glean3:03496 (Sp-Arf4/5) | intracellular | **2** | 9% | 2,10,13,  16 | 9.2E-18 | 0.5 |
| 09164 **^1(a)^** | Sp-Hsp701E/similar to heat shock protein 70 isoform 3; shares 1 peptide with Glean3:009165 (Sp-Hsp701A) and 1 peptide with Glean3:09477 (Sp-Hsp701C) | intracellular | **3 ^4^** | 5% **^4^** | 6 | 9.4E-73 | 0.5 **^4^** |
| 09922 **^1(a),(b)^** | Sp-Cys-rich_fgfr_1/similar to Golgi apparatus protein 1/similar to ENSANGP00000020376 (aa831-1085); domains: Cys-rich repeats; shares 3 peptides with Glean3:08505 (Sp-Cys_rich_fgfr) | membrane-  associated  (Golgi) | **6 ^4^** | 6% **^4^** | 10-14 | 2.9E-143 | 0.5 **^4^** |
| 04879 | Sp-Fam55cL_2/similar to protein of unknown function, partial | extracellular | **2** | 6% | 4-6,15 | 4.2E-78 | 0.4 |
| 14496 **^1c,d^** | Sp-Msp130r4; GPI | extracellular | **4** | 7% | 5,6 | 1.4E-47 | 0.4 |
| 15163 | Sp-Ccp/Hyr/7tm/Gpcr/similar to egg bindin receptor 1 | transmembrane | **4** | 6% | 3-9 | 3.7E-69 | 0.4 |
| 15537 **^1b^** | Sp-Arrdc2L; domain:Arrestin_N | intracellular | **4** | 14% | 1-8 | 1.5E-101 | 0.4 |
| 15763 | Sp-Msp130r5;GPI | extracellular | **3** | 5% | 5,6 | 1.6E-18 | 0.4 |
| 18348 **^1a^** | Sp-CasprL/similar to neurexin IV/similar to contactin-associated protein 5 | transmembrane | **3** | 6% | 1,3-5 | 7.8E-32 | 0.4 |
| 18529 | Hypothetical protein; domains: DM13, Domon | transmembrane | **3** | 5% | 4,5 | 8.1E-15 | 0.4 |
| 19034 **^1a^** | Sp-Hypp_925; domain: IG | transmembrane | **3** | 3% | 5-9,11,  13 | 7.9E-26 | 0.4 |
| 21824 | Hypothetical protein; domain: kringle | unknown | **2** | 14% | 1,3,7,  9-13 | 2.4E-42 | 0.4 |
| 23083 | Sp-Eif4A (translation initiation factor 4A) | intracellular | **2** | 7% | 1,10,14 | 1.0E-104 | 0.4 |
| 24019 **^1a,b^** | Sp-nephl/similar to nephrin;domains: IG, FN3; shares 3 peptides with Glean3:19665 (Sp-Hypp_941) | transmembrane | **5** | 5% | 1-7 | 1.5E-52 | 0.4 |
| 26703 | Sp-Hypp_2862; domains: IG | transmembrane | **2** | 6% | 13 | 1.7E-57 | 0.4 |
| 00437 | Sp-Z211 (zf-c2h2)/similar to megalin;domains: LDL receptor, EGF, zinc finger | extracellular/  transmembrane | **6** | 4% | 1-4,11 | 2.4E-70 | 0.3 |
| 01129 **^1b^** | Sp-Lrr/Igr_10 | transmembrane | **3** | 4% | 10,11,15 | 9.1E-12 | 0.3 |
| 08329 | Similar to KIAA0789 protein; domain: partial sulfotransferase_1 | unknown | **2** | 11% | 9,10,14,  15 | 3.0E-16 | 0.3 |
| 10644 **^1a,b^** | Sp-B3galt1_9 (galactosyl transferase) | transmembrane  (Golgi) | **2** | 6% | 13-15 | 2.0E-91 | 0.3 |
| 16020 **^1(a)^** | Sp-Enpep_2 (glutamyl aminopeptidase) | extracellular/  transmembrane | **2** | 5% | 3-5 | 7.3E-36 | 0.3 |
| 21193 | Sp-Adam/TS16/18L/similar to ADAM metallopeptidase with thrombospondin type 1 motif, 16 preproprotein | extracellular | **2** | 5% | 11-13 | 1.7E-17 | 0.3 |
| 22631 **^1(a),c^** | Sp-Hypp_2620/similar to TRIAD1 type I; domain:EMI; 19%Gly, 12%Pro | extracellular/  transmembrane | **2** | 6% | 1-13 | 1.1E-27 | 0.3 |
| 27172 **^1a,b^** | Hypothetical protein; domain: alpha/beta hydrolase | extracellular/  transmembrane | **2** | 9% | 12,13 | 2.3E-24 | 0.3 |
| 27850 | Sp-Glud1 (glutamate dehydrogenase 1); shares 1 peptide with Glean3:13010 (Sp-Glud1_1) and Glean3:27851 | intracellular  (Mito) | **2** | 4% | 1,3,7 | 3.6E-05 | 0.3 |
| 01929 | Sp-Exostl (exostosin-like) | transmembrane | **2** | 6% | 12,13 | 1.5E-66 | 0.2 |
| 22132  06165 | Sp-LrpL_5/similar to gp330/GH1281p/megalin  Sp-LrpL_1/similar to gp330/GH1281p/megalin  share the same set of peptides | transmembrane | **3** | 2% | 1-6 | 3.5E-38 | 0.2 |
| 11180 **^1a,b,c,d^** | Sp-Fn3/Egff_1; domains:FN3, EGF; extension-like protein; 16% proline | transmembrane | **2** | 4% | 9 | 5.5E-15 | 0.2 |
| 11877 | Sp-Ahi (integrin alpha subunit) | transmembrane | **2** | 2% | 4,5 | 6.3E-45 | 0.2 |
| 15906 **^1a,b^** | Sp-Hypp_120 | transmembrane | **4** | 3% | 11,14 | 1.8E-17 | 0.2 |
| 17605 ^1(a),b^ | Sp-Atp5a1 (ATP synthase, α subunit) | membrane  (mito) | **3** | 5% | 2,9,13 | 3.8E-10 | 0.2 |
| 19426 | Sp-Vps4B (vacuolar protein sorting factor 4B)/similar to MGC139102 protein | membrane-ass. (endosome) | **2** | 6% | 5,6 | 2.8E-07 | 0.2 |
| 20031 **^1a^** | Sp-Pam/similar to peptidyl-glycine alpha-amidating monooxygenase-B | transmembrane | **2** | 3% | 10,13 | 4.4E-05 | 0.2 |
| 20931 | Sp-Tm9sf2 (transmembrane 9 superfamily member 2); shares 2 peptides with Glean3:20427 (Sp-Tm9sf2_2) and 1 peptide with Glean3:10157 (Sp-Tm9sf2_1) | transmembrane | **2** | 5% | 10 | 1.7E-06 | 0.2 |
| 22588 | Sp-DNAse1_2 | extracellular/  intracellular | **2** | 4% | 7 | 3.8E-13 | 0.2 |
| 05193 | Sp-Tcp2/similar to C3 and PZP-like, alpha-2-macroglobulin domain containing 8 | extracellular/  membrane-ass. | **3** | 2% | 4,6,7 | 7.8E-09 | 0.1 |
| 05955 | Sp-Notchl1/similar to notch ligand | transmembrane | **2** | 1% | 1-5 | 9.0E-15 | 0.1 |
| 05999 | Sp-Fras1/similar to Fraser syndrome 1 | transmembrane | **5** | 2% | 3-7,9-11 | 5.2E-26 | 0.1 |
| 10829 | Sp-Ef2 (elongation factor 2) | intracellular | **2** | 3% | 2,10,14 | 8.1E-73 | 0.1 |
| 11588 **^1a,(b),d^** | Sp-Spc39L (solute carrier family 39)/similar to 2α fibrillar collagen; domains: KAZAL, Zip; | transmembrane | **2** | 1% | 1-14 | 1.1E-258 | 0.1 |
| 12786 | Sp-Bmp5/6/7/8 (bone morphogenetic protein) | extracellular | **2** | 2% | 13,14 | 2.1E-10 | 0.1 |
| 13889 | Sp-Pxdn (peroxidasin-like) | intracellular | **2** | 2% | 3-5 | 4.1E-25 | 0.1 |
| 24925 | Sp-Sfe1 (fertilization envelope component) | extracellular | **2** | 2% | 14,15 | 2.8E-40 | 0.1 |
| 26949 **^1a,b,c,d^** | Sp-Tf (transferrin) | extracellular | **2** | 2% | 3-5 | 2.0E-06 | 0.1 |
|  |  |  |  |  |  |  |  |

**^1a,b,c,d^**, protein identified previously in tooth powder matrix (**1a**), intact tooth matrix (**1b**) [26,27], test matrix (**1c**), or spine matrix (**1d**) [25,27]; brackets indicate tentative identification in the respective compartment. **^2^**, cellular location according to GO annotations in SpBase, predicted signal sequences, transmembrane sequences, non-classical secretion features, or similarity to known proteins. **^3^**, gel sections with > 5% of total peptides. **^4^**, sum of unique, razor, and non-unique peptides. The entries are ordered according to decreasing abundance. Additional data for identified proteins and sequences of unique peptides, scores, and more details see additional file 3 and additional file 4. GPI, predicted anchoring of extracellular proteins via a glycosylphosphatidylinositol anchor.
