# Supplementary material for: Proteomic analysis of sea urchin (Strongylocentrotus purpuratus) spicule matrix
Source: Proteome Sci. 2010 Jun 17;8:33. doi: 10.1186/1477-5956-8-33 (PMC2909932; doi:10.1186/1477-5956-8-33)
Supplement: Additional file 2 — Tentatively identified spicule matrix proteins. [file 1477-5956-8-33-S2.DOCX]

**Tentatively identified spicule organic matrix proteins.**

| **Glean3_ entry** | **Protein** | **Presumed**  **compart-**  **ment ^2^** | **Unique pep-tides** | **Sequen-ce cover-age** | **Gel section ^3^** | **Protein**  **Score**  **(PEP)** | **emPAI** |
| --- | --- | --- | --- | --- | --- | --- | --- |
|  |  |  |  |  |  |  |  |
| 15937 | Sp-Srcr128 (scavenger receptor-related) | transmembrane | **2** | 7% | 1-4 | 1.2E-05 | 2.7 |
| 17737 **^1(a),b^** | Hypothetical protein , limited similarity to ECM18 of *L*. *variegatus*; 12% Cys; domain: MEROPS inhibitor family I8 | extracellular | **2** | 26% | 1,13-15 | 6.7E-13 | 2.2 |
| 07494 | Hypothetical protein; domain: thrombospondin type 1. ADAMTS | extracellular/  transmembrane | **2** | 21% | 1,2,14 | 6.8E-06 | 1.2 |
| 01114 | Hypothetical protein similar to apextrin; domain: membrane-attack complex/perforin (MACPF) | extracellular/  transmembrane | **2** | 9% | 6,7 | 9.2E-07 | 0.5 |
| 01489 | Sp-CalcneurL-2 (calcineurin-like acid phosphatase) | extracellular | **2** | 3% | 8 | 6.1E-05 | 0.4 |
| 11039 | Sp-Gage1 (endo16 calcium-binding protein) | extracellular | **2** | 5% | 10,12,14 | 1.9E-09 | 0.3 |
| 11352 | Sp-Rps3A (ribosomal protein S3a); shares 1 peptide with Glean3:01585 (Sp-Pdrps3a) and Glean3:20308 (Sp-Rps3A_1) | intracellular | **2** | 6% | 10,14 | 3.2E-05 | 0.3 |
| 22561 **^1d^** | Sp-Mipphp1 (multiple inositol polyphosphate histidine phosphatase 1) | extracellular (ER) | **2** | 5% | 6,7 | 7.7E-6 | 0.3 |
| 13368 | Sp-Prdx6/similar to peroxireductase | intracellular | **2** | 4% | 1,10,14 | 3.2E-05 | 0.2 |
| 28366 | Sp-Cask_1 (calcium/calmodulin-dependent Ser/Thr kinase; ~aa160-516); ~aa1-160 ribosomal protein P0 ; | intracellular/  membrane | **2** | 6% | 10,14 | 7.5E-06 | 0.2 |
| 16181 | Sp-Lrp4-3/similar to Low-density lipoprotein receptor-related protein 4 | transmembrane | **2** | 2% | 5,13 | 4.1E-07 | 0.1 |
| 25815 | Sp-Atp1a3/Na/K-ATPase α-subunit | membrane | **2** | 3% | 4 | 1.0E-06 | 0.1 |
| 26008 **^1c,d^** | Sp-FcolI/II/III/similar to collagen α1 | extracellular | **2** | 1% | 11 | 1.5E-18 | <0.1 |
|  |  |  |  |  |  |  |  |

**^1a,b,c,d^**, protein identified previously in tooth powder matrix (**1a**), intact tooth matrix (**1b**) [26,27], test matrix (**1c**), or spine matrix (**1d**) [25,27]; brackets indicate tentative identification in the respective compartment. **^2^**, cellular location according to GO annotations in SpBase, predicted signal sequences, transmembrane sequences, non-classical secretion features, or similarity to known proteins. **^3^**, gel sections with > 5% of total peptides. **^4^**, sum of unique, razor, and non-unique peptides. The entries are ordered according to decreasing abundance. Additional data for identified proteins and sequences of unique peptides, scores, and more details see additional file 3 and additional file 4.
