# Supplementary material for: Proteomic analysis of sea urchin (Strongylocentrotus purpuratus) spicule matrix
Source: Proteome Sci. 2010 Jun 17;8:33. doi: 10.1186/1477-5956-8-33 (PMC2909932; doi:10.1186/1477-5956-8-33)
Supplement: Additional file 6 — Alignment of CTLLD sequences of matrix proteins. This file shows a ClustalW2 alignment of the sequences of predicted CTLLD enlisted in Table 1 (A) and a tree representation for graphical visualization (B). [file 1477-5956-8-33-S6.DOCX]

**A. ClustalW2 alignment of spicule matrix C-type lectin-like domains**

00826: SM30-B 45 TRTEACAKFWVQ--EGNSCYLFDSGAFLRQ--------------------------VAAS

00827: SM30-C 45 TRTEVCAKFWVQ--EGNSCYLFDSGAFLRQ--------------------------VAAS

00825: SM30-A 61 KRTETCAKFWVH--EGNSCYLFDSGAFLRQ--------------------------VTAS

04867: SM30-E 291 NRKERCAKFWME--EGNSCYLFDSGAFLRR--------------------------VAAN

04869: SM30-F 122 NGPGKCRPKWTE--YKGHCYLYSSGAVEQV--------------------------GNPV

00828: SM30-D 118 PTAISCPELWIQ--HKGSCYRMESGASSQMR-------------------------FGAT

00164: Sp-Clect ------------------------------------------------------------

18811: SM50 15 ATGQDCPAYYVRSQSGQSCYR---------------------------------------

18810: SM32 15 ATGQDCPAYYVRSQSGQSCYR---------------------------------------

05991: Sp-Clect_14 27 YPMEGCIPGWTN--FGKYCYK---------------------------------------

05989: Sp-Clect_13 28 YPLQGCQPGWTN--FGKFCYKPPQEKAESSSPKISPHTAHEPELKGGRAPWGKVTKVQIY

05990: SM29 101 APLAGCLPGWTN--FGNFCYK---------------------------------------

05992 17 ALAVTCPAGWAT--FGYSCYK---------------------------------------

11163: Sp-Clect_25 12 LAVTACPFGWSQ--SGRSCYK---------------------------------------

18813: SM37 16 AVQAQCPPSWVK--VENSCYR---------------------------------------

PM27 67 VGGSKCKGGWFL--IGQQCFK---------------------------------------

13825: Sp-Clect_76 12 YSLRVCIPRISH------------------------------------------------

27906: Sp-C-lectin/PMC1 16 SALPQCPEGWTY--SLKKCYK---------------------------------------

00826: SM30-B 77 RPVVVNNENGLFQAAANMYCGQMHPNAS-------------LVTVNSLAENNFLYEWAVR

00827: SM30-C 77 RPVVVNNQDGLFQAAANMYCGQMHPNAS-------------LVTVNSLAENNFLYEWAVR

00825: SM30-A 93 GPLIVNNENGLFQAAANMYCGQMHPGAN-------------LVTVNSLTENNFLYEWAVR

04867: SM30-E 323 APIVVNNQDGLFQAAANTYCSQMHPNAS-------------LVTVNSLAENNLLYEWAVR

04869: SM30-F 154 THLGVNAQEGKTQDAANLFCASQQIGAT-------------LVTLNDVEENDFLYSWAVD

00828: SM30-D 151 GPTFYGYNEGLTQASANSYCGVLQPGSS-------------LVTVNTLEENNFLYKWVVG

00164: Sp-Clect 14 -----IRLEGMPFAQANLFCGQCHPGAH-------------LVVISDAAEHEFLYNNFYG

18811: SM50 36 -----YFNMRVPYRMASEFCEMVTPCGN----GPAKMGALASVSSPQENMEIYQLVAGFS

18810: SM32 36 -----YFNIPLAYQWASEFCEMVTPCGN----GPAVMGTLAAPKSPQENMEIYRLVASFS

05991: Sp-Clect_14 46 -----FFNQRMNWLQAQRECNKYSALNG--------TTPRGYLMTSKDQQHNNFQHNWLR

05989: Sp-Clect_13 86 VQEARYFYERLPFMEAQMQCLSFRASP---------LAPRGYLITAKDQTHNRFQHNFLT

05990: SM29 120 -----YIWQRLPYDEANMFCARFSAQAVPGQRPGFGMPPRGHLIVTKDMMHNKFQHNWLR

05992 ------------------------------------------------------------

11163: Sp-Clect_25 31 -----VSQMAATYEQAAMLCSQQSSCETG---------FGGHLVSITTDFEKQQVTDILN

18813: SM37 35 -----AFGQLKTYDMASQSCKTFTGCDG--------ISPGHLAAPTTFEERRAVRKIRFD

PM27 86 -----MMSRALKWNDAELMCEQNAPCGTP--------VLGGVMTIPDIQTSNAVINHLKS

13825: Sp-Clect_76 24 -------RIRMTFNEATFFCNRYGGSLY---------------ALDSPSKNRIMLSYLTT

27906: Sp-C-lectin/PMC1 35 -----FFPMPLIKEEATVACTEAPNVIGG--------KLLDIADFDSFRFVKNLYTHTLP

00826: SM30-B 124 MMVE-------------PEPVWIGLHAG---PMG-QWQWYSG-EPVTYTN------WERM

00827: SM30-C 124 MMVE-------------PEPVWIGLHAG---PTG-LWQWYSG-EPVTYTN------WERM

00825: SM30-A 140 MMVE-------------PVPVWIGLHVG---PTG-LWQWYSG-EPVTYTN------WEGM

04867: SM30-E 370 MMVE-------------PVPVWIGLHVG---PMG-RWQWYSG-EPVNYTN------WEGF

04869: SM30-F 201 MMVE-------------PVPVWIGLHAHPT-PTGYVWQWFDEKEPVLFTN------WDRP

00828: SM30-D 198 MLGHN------------ARPVWIGLHVG---PTG-LMQWYSG-ETSAYTN------WEEI

00164: Sp-Clect 56 FEPT---------------EVWVGLFFN---STTLAWEWVNG-EPALNVP------WEPF

18811: SM50 87 QDNQM------------ENEVWLGWNSQ------SPFFWEDG-TPAYPNG---FAAFSSS

18810: SM32 87 QDNQM------------EREVWLGWNSM------NPFMWENG-APAYPHG---FSAFDSG

05991: Sp-Clect_14 93 YTGGG------------LNKVWLGLSEK----TNNNYFWADG-TPLLPTQ---WNKWKID

05989: Sp-Clect_13 137 FTGGA------------NNKVWMGLAER----QYNVFWWADG-TPFRPHD---WNLFKPD

05990: SM29 175 YTGGG------------ANKIWMGLAELPSAPESNRYFWADG-TEFFFTRNFVFNRFKPD

05992 36 ------------------------------------YQWIDH-TPVAYTN------WKVD

11163: Sp-Clect_25 77 FNQMA------------RNETWLGLRTYN-----GPLRWASG-EPLNPALTN---PWHPG

18813: SM37 82 LLRPIGGNRIGEGQRLRPSNVWMGFRLGQG--ANSSWDSTEQYDNLLLDQ-----HWGPN

PM27 133 LSSTAMA---------IDIPFWTGLHNKWN----ALLERYEG--WKWPAG------WSTT

13825: Sp-Clect_76 62 VFGQG-----------RRQKFWTGFYKAEN---ALGWRWSTG-STSQYTD------WEAG

27906: Sp-C-lectin/PMC1 82 FYQP-------------PTGQQLDFIPP------EKAIWTALAFDWNLEQ------WTTG

00826: SM30-B 160 T-APMAEPGLGAMIFDADIIAQMFNNQVEITP-----------------------QWVPE

00827: SM30-C 160 T-APIAEPGLGAMIFDADIIAQMFNNQVEITP-----------------------QWVPE

00825: SM30-A 176 R-VPQAEPGLGAMIFDAEIANQVFNNQVEIAP-----------------------QWVPE

04867: SM30-E 406 R-APMAEPGLGAMIFDADIINQIFNNQVEITP-----------------------QWVPE

04869: SM30-F 241 P-APVARAGLGAVLFDADLT-VVPPNAIEISG-----------------------KWLPV

00828: SM30-D 235 P-EPFD----GATMFDVQPNNQMNN-QVDLTS-----------------------QWSRE

00164: Sp-Clect 91 YGEPLVTHQGAAMMYVSEVIDQQTTLKYGYGI-----------------------EVSDY

18811: SM50 125 PASPPRPGMPPTRSWPVNPQNPMSGPPGRAPVMKRQNPPVRPGQGGRQIPQGVGPQWEAV

18810: SM32 ------------------------------------------------------------

05991: Sp-Clect_14 133 Q---------PQLNAHIQGVHTFDNMVDMT--------------------------WVTS

05989: Sp-Clect_13 177 Q---------PQQNHHIDGVFTFDNYPYQT--------------------------WVTS

05990: SM29 222 Q---------PQQNAHRQAVHSFNNRPDNS--------------------------WVTT

05992 53 Q---------PQLNWHRQAAHIFNGRELQT--------------------------WVTS

11163: Sp-Clect_25 116 N----------PMRGPNMCTVSFQN------------------------------RWATR

18813: SM37 135 R--PNMPGQRPQAQAFDPTNFRWNGRCMTLPG--------------QMQMNGAVQKWGHI

PM27 238 Q---------QPLRFVNWAPREPNNQLLDQQH-----------------------SYCAR

13825: Sp-Clect_76 101 K---------PATTNEAIGAVAWGGTHHWSDT------------------------WQDE

27906: Sp-C-lectin/PMC1 117 T----TGTTGTPGTTPAEPFYRFNNFNSQPIG-------------------------KPP

00826: SM30-B 196 QAI---NDRHALICEYHPS

00827: SM30-C 196 QAI---NDRHALICEYHPS

00825: SM30-A 281 EAI---NDRHSIICEYHPS

04867: SM30-E 442 QGR---NEPHALICEYHPQ

04869: SM30-F 276 EGLGPQAEPLGLICEYSLH

00828: SM30-D 266 DPY----NERMFICEHRPR

00164: Sp-Clect 128 LVSELGEEPLPFICEYSLN

18811: SM50 185 EVT----AMRAFVCEVPAG

18810: SM32 -------------------

05991: Sp-Clect_14 158 FYK----TEMSFICQYQYM

05989: Sp-Clect_13 202 SHE----TQMSFTCQYQYL

05990: SM29 247 SVE----TEVSFICQYQYK

05992 78 NWR----TPMSYVCKLRFG

11163: Sp-Clect_25 136 NCN----MRRQFICEMGGS

18813: SM37 179 ACN----MQAAYMCEISPA

PM27 200 MNRMG--QWYVVRCDEPMY

13825: Sp-Clect_76 128 PLS----ASFAFICQAPAL

27906: Sp-C-lectin/PMC1 148 AEP-----LELLSCTVYPA

----------------------------------------------------------------------------------------------------------------------------------------------------------------------------------------------------

This alignment was done with ClustalW2 as provided by the European Informatics Institute (EBI) available at <http://www.ebi.ac.uk/Tools/clustalw2/index.html> with default settings. The most similar pair is on top. C-type lectin-like domains (CTLLD) were predicted using InterProScan (<http://www.ebi.ac.uk/Tools/InterProScan/>). The sequences were chosen to start five amino acids in front of the first Cys and to stop five amino acids after the last Cys of the typical 4-Cys motif. If sequences contained an incomplete motif, only residues predicted to belong to the CTLLD were used. Red, hydrophobic residues A, V, F, P, M, I, L, W; blue, acidic amino acids D and E; magenta, basic amino acids K and R; green, hydrophilic amino acids S, T, Y, H, C, N, G, Q. Conserved cysteines identified as part of the CTLLD are shaded yellow. The clustalW2 dnd file was used to produce the cladogram shown below.

**B. Tree representation of sea urchin skeletal matrix C-type lectin-like domain alignment**


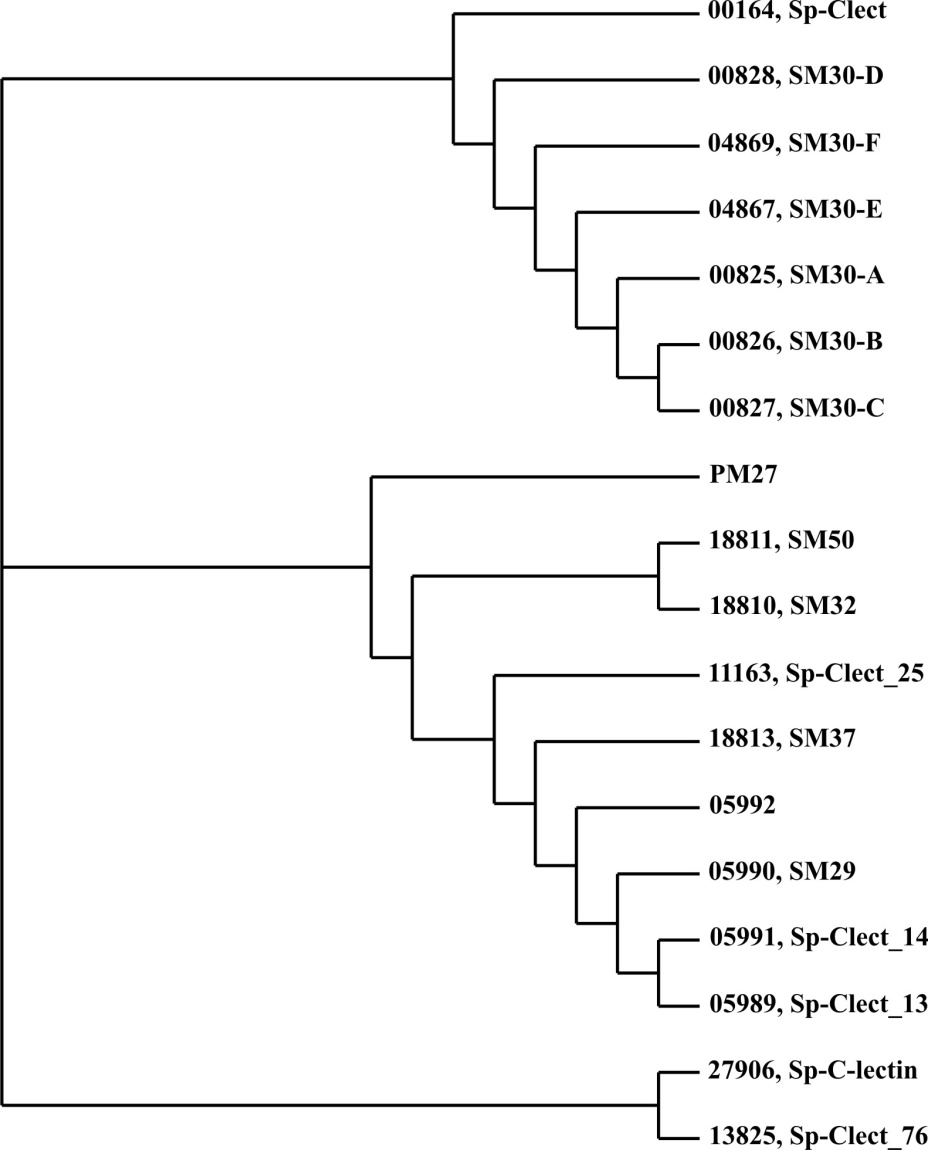


----------------------------------------------------------------------------------------------------------------------------------------------------------------------The dnd file of clustalW2 was fed into the treeview program (<http://taxonomy.zoology.gla.ac.uk/rod/treeview.html>), described in Page RDM: **TREEVIEW: An application to display phylogenetic trees on personal computers.** *Computer Applications in the Biosciences* 1996, **12**:357-358, to produce this rectangular cladogram. Branch lengths are proportional to inferred evolutionary change.
