# Supplementary material for: Proteomic analysis of sea urchin (Strongylocentrotus purpuratus) spicule matrix
Source: Proteome Sci. 2010 Jun 17;8:33. doi: 10.1186/1477-5956-8-33 (PMC2909932; doi:10.1186/1477-5956-8-33)
Supplement: Additional file 7 — Selected MS/MS spectra of SM30-D peptides. This file contains a ClustalW2 alignment of SM30-D sequence variants and MaxQuant-annotated MS/MS spectra of unique SM30-D peptides. [file 1477-5956-8-33-S7.DOCX]

SPU_000828 MQGFVCVLVC-LAALAAQTQGQVGQPGG--QPGRQPGR**QPTGQPTGQPR**MQPTGQPRQPG 57

Glean3_00828 MQGFVCVLLLKLKGRLDNREDNRGDNRGDNRLDNRLDNRVCNRLDNHDNLEDNRDNRADN 60

********: * . : :.: *: * : ..: ..: .: .: .:: . : * .

SPU_000828 **RQPGQPGGQPGGQPGGQPGFPGGQPGLPGGQPEFP-R**SQ--------------------P 96

Glean3_00828 REGNREDNR--GFPVDNPDYPVDNQNFRVRNQDFRVRNQDNRVDSPVVNPVSAQLLYHVP 118

*: .: ..: * * .:*.:* .: .: : :* *.* *

SPU_000828 GFPR-**SQPGQPGGQPGGQPGIRPTAISCPELWIQHK**GSCYR**MESGASSQMR**FGATGPTFY 155

Glean3_00828 NFGYNTRAAAIGNFFQGQGVNFLALIQVPGSSSYGTGSRNC**MESGASSQMR**FGATGPTFY 178

.* ::.. *. ** : *. * .** *******************

SPU_000828 GYNEGLTQASANSYCGVLQPGSSLVTVNTLEENNFLYK**WVVGMLGHNAR**PVWIGLHVGPT 215

Glean3_00828 GYNEGLTQASANSYCGVLQPGSSLVTVNTLEENNFLYK**WVVGMLGHNAR**PVWIGLHVGPT 238

************************************************************

SPU_000828 GLMQWYSGETSAYTNWEEIPEPFDGATMFDVQPNNQMNNQVDLTSQWSREDPYNER**MFIC** 275

Glean3_00828 GLMQWYSGETSAYTNWEEIPEPFDGATMFDVQPNNQMNNQVDLTSQWSREDPYNER**MFIC** 298

************************************************************

SPU_000828 **EHR**PRGLGAPAPTQPGATMRPFMLSNNRNSLMGVLRGGAFGGSRLQEVRRGRPASFRMNP 335

Glean3_00828 **EHR**PRGLGAPAPTQPGATMRPFMLSNNRNSLMGVLRGGAFGGSRLQEVRRGRPASFRMNP 358

************************************************************

SPU_000828 YFAVR- 340

Glean3_00828 YFAVRP 364

*****

-----------------------------------------------------------------------------------------------------------------------------------------------------------------------------------------------------

**Comparison of SM30-D sequences contained in our Glean3 database and the actual SpBase databases.**

Clustal alignment of the sequences shows that they differ in the N-terminal third, i.e. the part containing Pro-rich repetitive sequences. The C-terminal containing the CTLLD is identical in both sequences. MS/MS-sequenced peptides are shown in bold red and confirm the SpBase sequence (SPU:000828). **Spectra of unique peptides are shown in Fig.3 and below.**


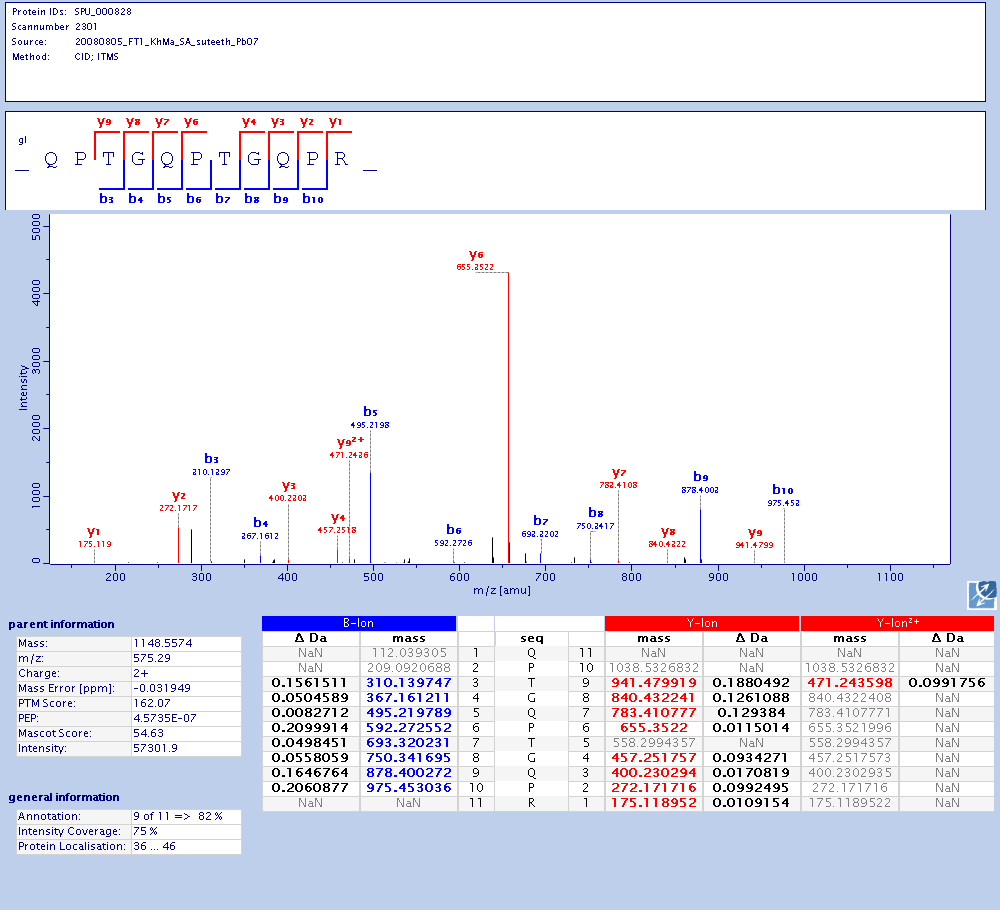


Concordantly with fragmentation rules the most intense fragment, y6, results from a cleavage in front of proline. Almost complete y- and b-ion series allow the unequivocal identification of this peptide.


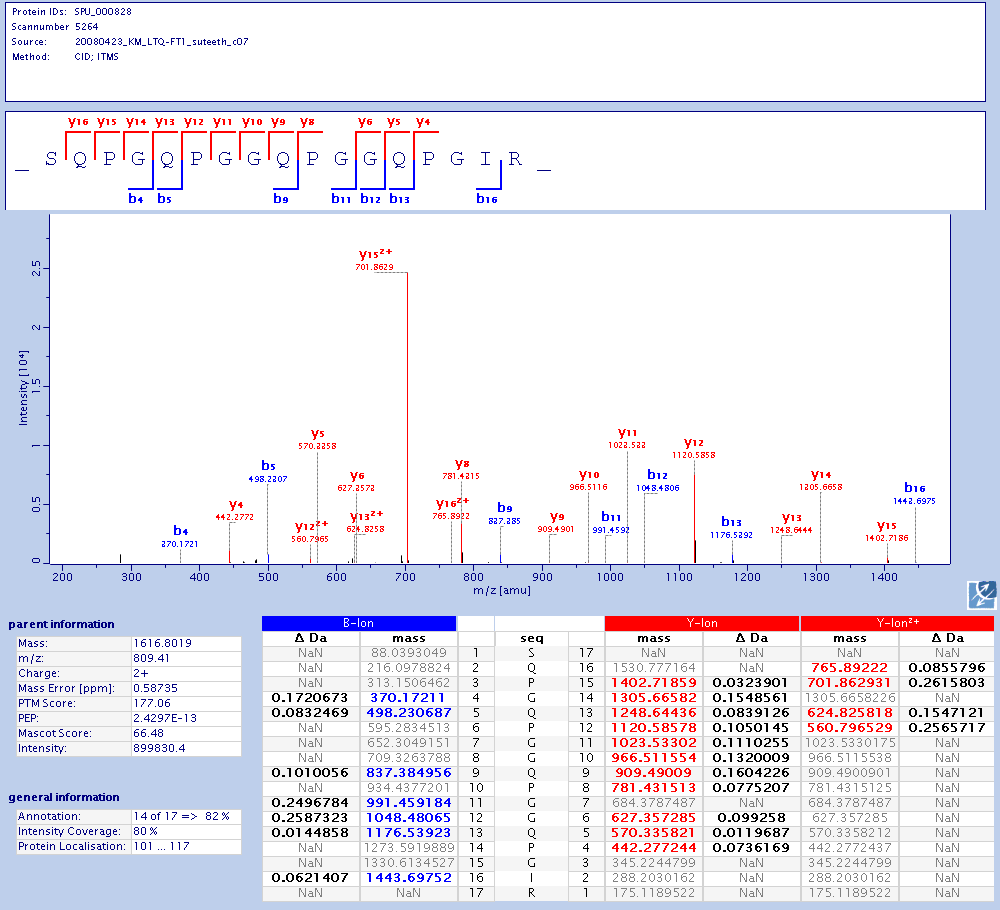


As in the previous spectrum the most intense fragment, y15 2+, is derived from a cleavage in front of a proline. A long uninterrupted series of y-ions, supported by a shorter series of y-ions and b-ions, is the basis for high PEP and Mascot scores.


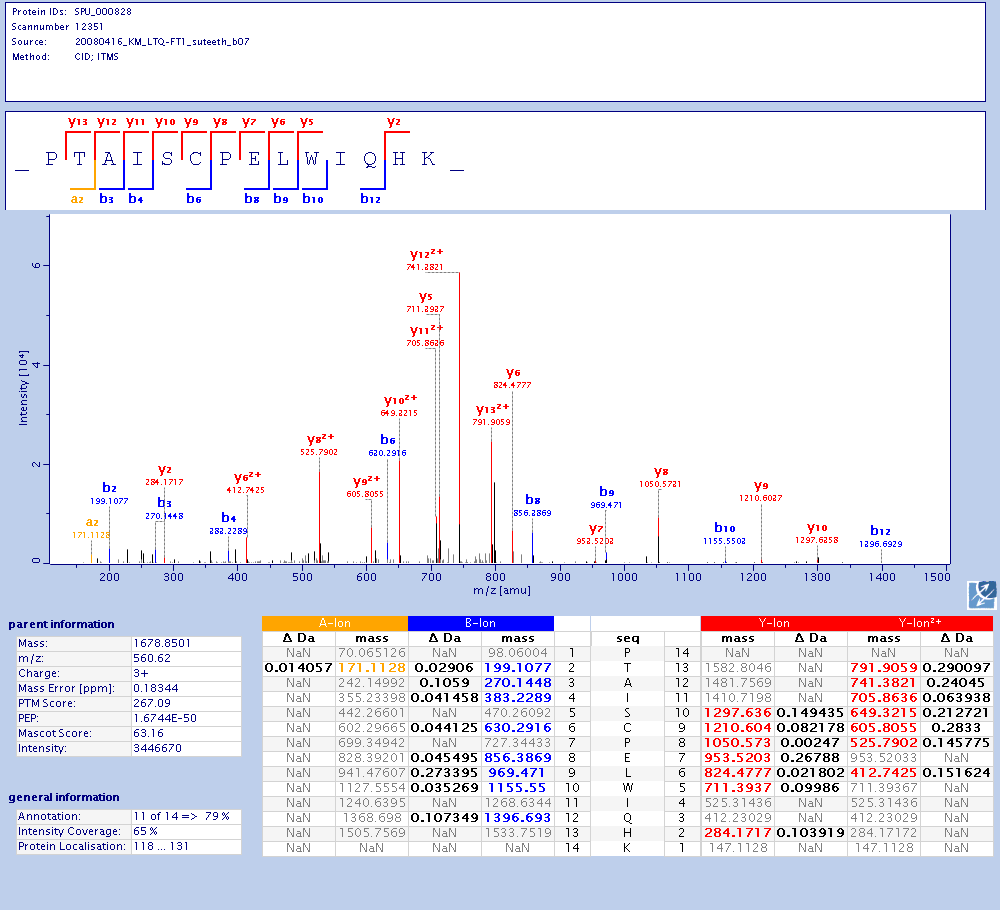


This peptide is triply charged in agreement with the presence of an internal positively charged residue (His) in addition to the C-terminal Lys. The presence of an a2/b2 pair and an extended series of y-ions supported by several b-ions yields high PEP and Mascot scores for this peptide, although there is no preferential cleavage N-terminal to proline.


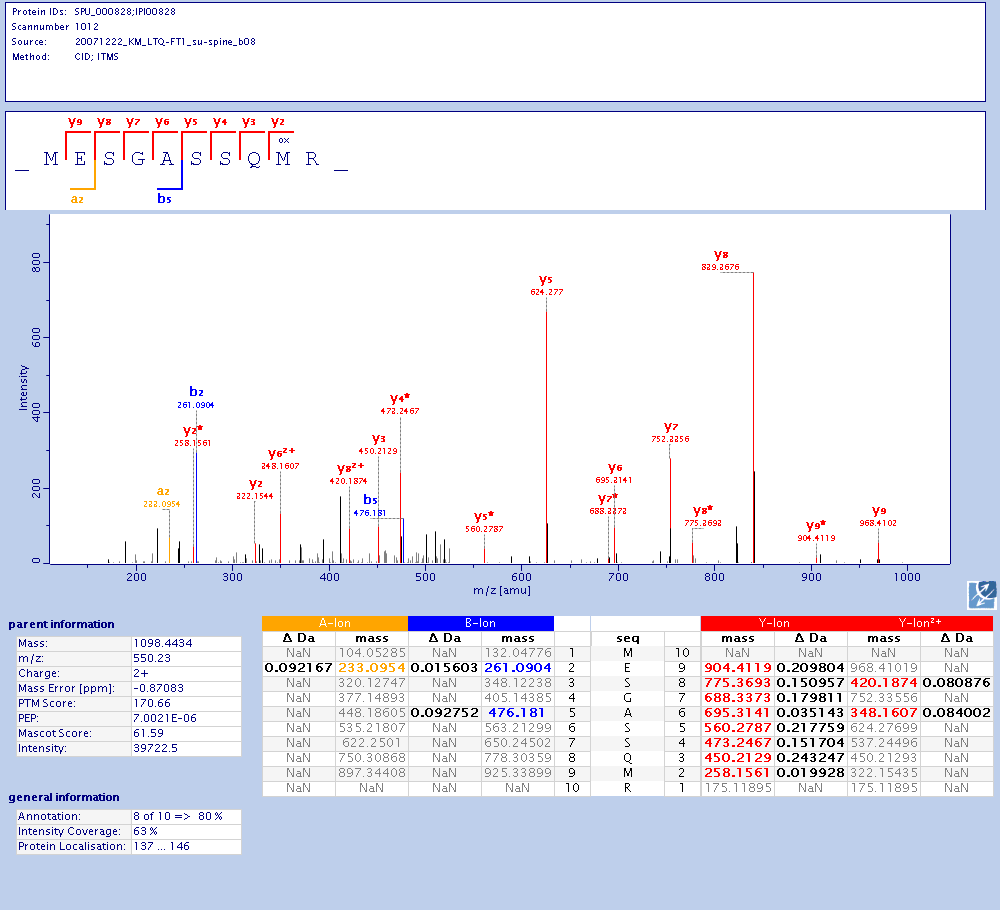


* indicates loss of SOCH_4_ (-64Da for singly charged fragments) from the oxidized Met in several y-ions. An a2/b2 pair is present in addition to an almost complete y-ion series. The relatively intense unassigned peak in front of the y8^2+^ ion at a n m/z of 411.3604 most probably results from loss of water from y8^2+^ ([y8-H_2_O]^2+^ , m/z=411.1822).


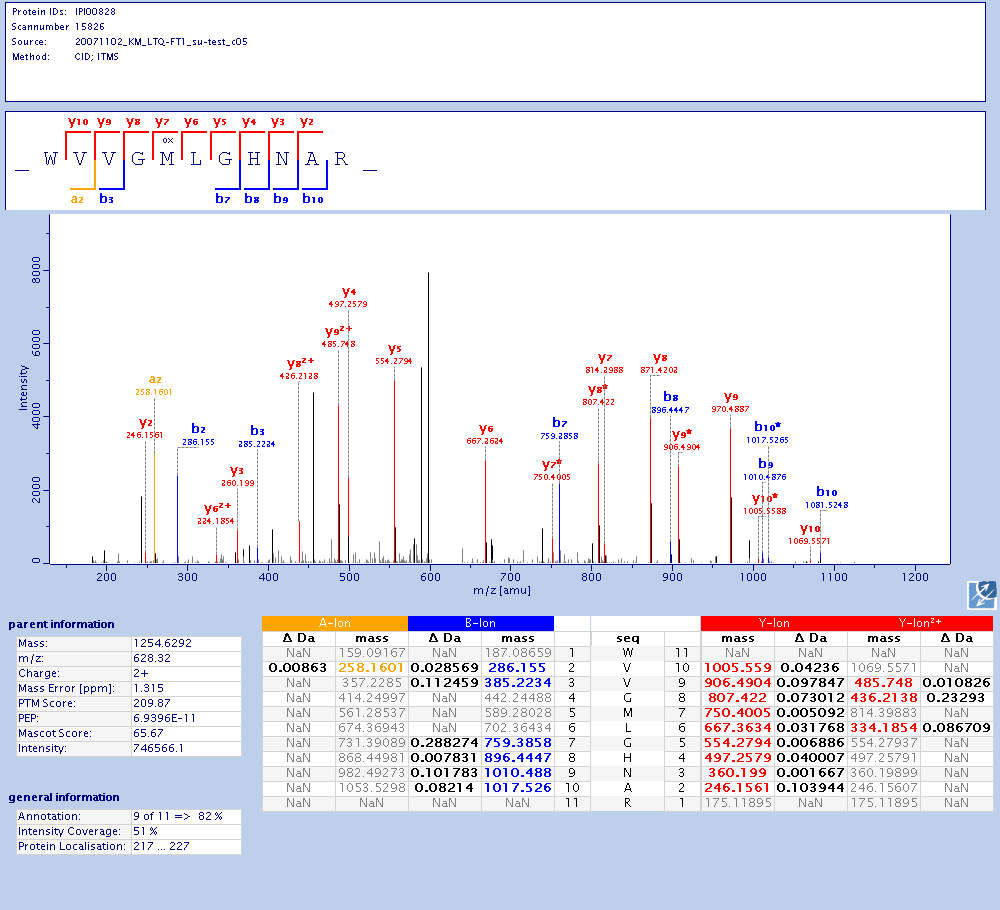


Fragmentation of a doubly charged parent ion of this peptide. * indicates loss of SOCH_4_ (-64Da for singly charged fragments) from the oxidized Met in several y-ions starting with y7 and b10, in agreement with the position of the oxidized Met. The major non-annotated peak at m/z 596.470 most probably represents the MH^2+^ peptide ion showing loss of SOCH_4_ (-32Da from a doubly charged ion) with a calculated m/z of 596.3227. The major non-annotated peak at m/z 453.905 is most probably the [y9-SOCH_4_]^2+^ ion (theoretical m/z 453.749). Recognition and annotation of doubly charged ions with losses of this type was not implemented in the MaxQuant version we used (1.014.6), but is implemented in more recent higher versions. A triply charged form of this peptide, caused by the presence of an internal basic residue (His), was also detected.


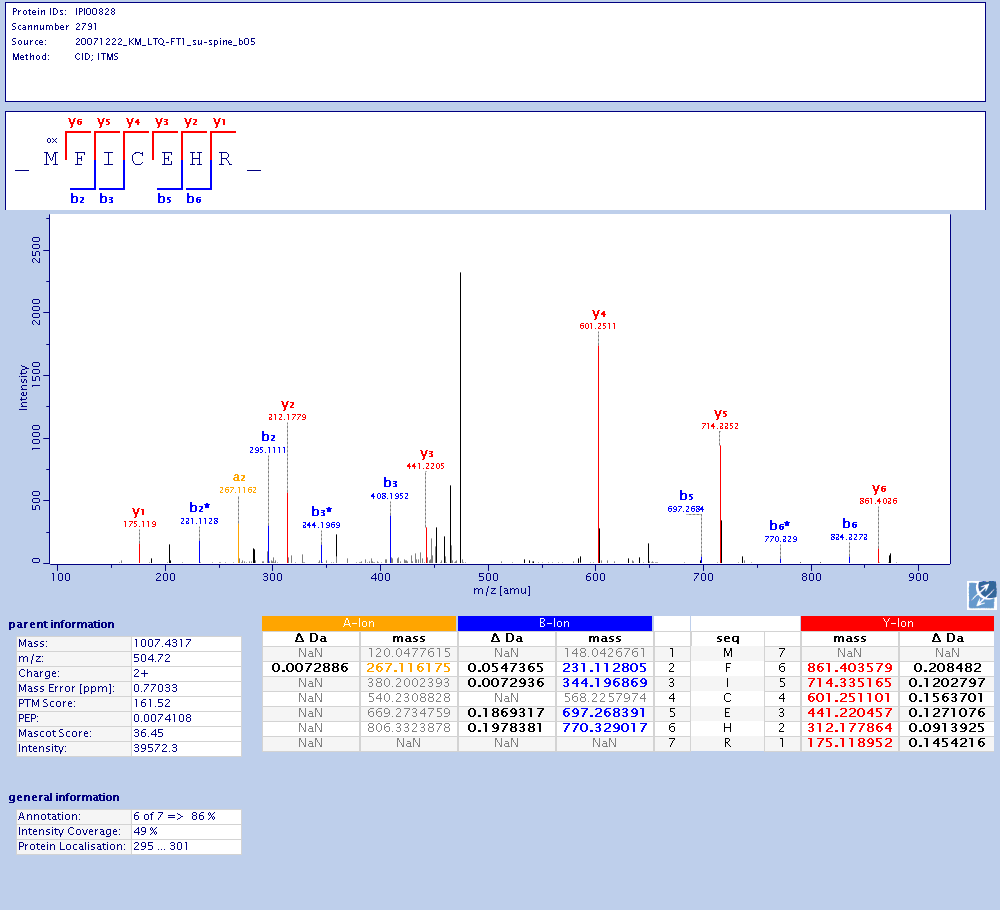


* indicates loss of SOCH_4_ (-64Da for singly charged fragments) from the oxidized Met in several b-ions. The major non-annotated peak at m/z 472.976 is most probably the MH^2+^ peptide ion showing loss of SOCH_4_ (-32Da from a doubly charged ion) with a calculated m/z of 472.724. The less intense peak in front of the [MH-SOCH_4_]^2+^ at m/z 464.123 may be the same with loss of H_2_O from Glu (-8.853Da as compared to a theoretical value of 9Da for a doubly charged ion).
