# Supplementary material for: Proteomic analysis of sea urchin (Strongylocentrotus purpuratus) spicule matrix
Source: Proteome Sci. 2010 Jun 17;8:33. doi: 10.1186/1477-5956-8-33 (PMC2909932; doi:10.1186/1477-5956-8-33)
Supplement: Additional file 8 — Alignment of Sp-Mmp18/19-like sequences. [file 1477-5956-8-33-S8.DOCX]

09924 ------------------------------------------------------------

09925 MPRLVVLLLGCCLAAVGSMAAEPIETQEQIWTFMKKYGYITENDMVNGMPKDEETKTKSI 60

05577 ------------------------------------------------------------

05723 ------------------------------------------------------------

09924 ------------------------------------------------------------

09925 TYFQKMGNMTMTGTLDEESMELMNTPRCGMKDMESFADMMRRKRYALGPRWR**QTDLTWNI** 120

05577 ------------------------------------------------------------

05723 ------------------------------------------------------------

09924 ---------------MARAFKAWSDVSSLTFRK**VTTNEPDIK**IIFALGDHGDGFEARFDG 45

09925 **LEDSDDLPR**EQVESIMARAFK**AWSDVSTLTFR**K**VTTNEPDIK*IIFAAGEHGDGIEAR***FDG 180

05577 ------------------------------------------------------------

05723 ------------------MVV**AWSDVSTLTFR**K**VTTNEPDIK*IIFAANEHGDGFNAR***FDG 42

09924 PGGVLAHAYFPSSSSLGGDAHFDEGEMFTEGTSRGVNLFQVAAHEFGHSLGLEHSDVEDA 105

09925 GGGVLAHAYFPTSNSLGGDAHFDEGERFTEGTSTGINLFQVAAHEFGHSLGLR**HSDIEDA** 240

05577 --------------------------MFTEGTSTGINLXQVAAHEFGHSLGLR**HSDIEDA** 34

05723 SGGVLAHAYFPTSNLIGGDAHFDEDETYTDGTSSGINLFQVAAHEFGHSLGLRHSDVQDA 102

09924 LMFPYYR**GYVPNFQLHR**DDIAGIQAQYGEGNGEPEEPNTPSTPLDNCMPQISLATRTEDG 165

09925 **LMFPYYR*GYVPNFQLHR***DDIAGIQAHYGEGNGQPEEPDTPSTPLDNCMPQISLATRTEDG 300

05577 **LMFPYYR*GYVPNFQLHR***DDIDGIQAHYGEGNGQPEEPDTPSTPLDNCMPQISLATRTEDG 94

05723 LMYPYYRGYVANFQLHRDDIAGIQAQYGEGNGQPEEPDTPSTPLDNCMPQISWATRTRDG 162

09924 SAYFANETHVFRRASNNTIPAGYPKRIGDEFPGLPTNLDAAIFYSPYTYFFKGSQYWRFR 225

09925 SAYFANETHVFRRTSNNIIPAGYPKRIGDEFPGLPTNLDAAIFYSPYTYFFKGSQYWR**FQ** 360

05577 SAYFANETHVFRR**TSENIIPAGYPK**RIGDEFPGLPTNLDAAIFYSPYTYFFKGSQYWR**FR** 154

05723 SAYFANETHVFRRASNNIIPAGYPKMIGDEFPGLPTDLDAAFYYSPYTYFFKGSQYWRFR 222

09924 **GQEMAGTYPR**PMSDWR**GVPTDVDSAFVWSR**NGGIYFTKGNEYYR**YTGQESSFYPR**PLSQF 285

09925 **NQEMAGTYPR**PMSDWR**GVPTDVDSAFVWSR**NGGIYFTKGNQYYRYTGRENSFYPQPLSHF 420

05577 **DQEMAGTYPR**PMSAWR**GVPTDVDSAFVWSR**NGRIYFTKGDQYYR**YTGQESSFYPQPLSHF** 214

05723 NRAMAGTYPRPLSDWQGLPNDLDSAYVWTRNGGIYFTKGNQYYRYNRGVSSYYPQPLSRW 282

09924 RGLPSDGVDAAFQYSNSITYFFKGSDYYRFNDSTVMVDNGYPLNTAIQWLGCDPNELLG- 344

09925 RGLPSDGVDAAFQYSNSITYFFKGSDYYRFNDSTVMVDNGYPLNTAIQWLGCDPNELLG- 479

05577 **R**GLPSDGVDAAFQYSNSITYFFKGSDYYRFNDRAVEVQSGYPLNTAIQWLGCDPNELL-- 272

05723 GGLPSDGVDAAFRYNNGYTYFFKGSEYYRFNDR**NINVASGYPR**NTAIQWLGCDPNELFLG 342

09924 --------PPADTGGDATVMVPSMVAVLFSALSAIYYAF 375

09925 --------PPADTGGDATVMVPSMVAVLFSALSAIYYAF 510

05577 -------------LGDATAMVPSMVAVLFSALSALYYAF 298

05723 PTADTGGDATADTGGDATVMVPSMVAVFFSALSALYYAF 381

**ClustalW2 alignment of Sp-Mmp18/19-like sequences identified in spicule matrix.** Residues identical in at least three sequences are shaded yellow. Peptides identified by MS/MS are in red. Peptides located at identical positions in the protein sequences but differing in sequence are underlined. These peptides validate different sequence.
