# Supplementary material for: Proteomic analysis of sea urchin (Strongylocentrotus purpuratus) spicule matrix
Source: Proteome Sci. 2010 Jun 17;8:33. doi: 10.1186/1477-5956-8-33 (PMC2909932; doi:10.1186/1477-5956-8-33)
Supplement: Additional file 9 — Comparison of thombospondins Sp-Thsd7b and Sp-Thsd7b_2. [file 1477-5956-8-33-S9.DOCX]

Glean3_19739 ----MAKTLKKTENSLTDVLS-----------VMAIVNDDMIYSFPSSE--EELLPSQSY 43

Glean3_25454 DWQQCRVTLNSSATCGAGIETR**QVFCVDGGSDVLTPVADELCFNSDSASLFKPVANR**ACY 60

**:.: .. :.: : *:: * *:: :. *:. : : .*

Glean3_19739 TQS---------RQKRSSSGSSSDGTPPERKRRNKTS-EHSAENSDAAIEQKC------- 86

Glean3_25454 LDCPVDCEVGHWSHWSSCTKSCGEGAVQMRYR**EVLVENVFGGQECPSQVELRACEAIECA** 120

:. : *.: *..:*: * *. .. ...::. : :* :.

Glean3_19739 ---QGRWTKCFLDRGYDDCGPGTRHR**AVYCMNALNK**RVDASYCAGKTKPDRQGTCRVPCA 143

Glean3_25454 **WWHTGR**WTKCFLDRGYDDCGPGKRHR**AVYCMNALNK**RVDASYCAGKTKPDRQGTCSVPCA 180

******************.******************************** ****

Glean3_19739 EDCVVSEWSDWGACSVSCGPNGGARKRSRR**IVAYPYNRDLNPCLSEDELTQSK**PCNIHVS 203

Glean3_25454 EDCVVSEWSDWGACSVSCGPNGGARKRSRR**IVAYPYNRDLNPCLSEEELTQSK**PCNIHVS 240

**********************************************:*************

Glean3_19739 CHTYSWQATAWGECQMNSTANCEEGVTGVGGCLMNETASCGERIGMETRDVGCEMETGSA 263

Glean3_25454 CHTYSWQATAWGECQMNSTANCGGGGFGIQ---RYDAVSKQNRSTSHKHN-----EEGSV 292

********************** * *: ::.* :* ..:: * **.

Glean3_19739 ADASQCDGWSLPEGSRPCDVACPQDCELSPWSTWSSCSASCGLHARRTRTKRVLTVPVNG 323

Glean3_25454 ADASQCDGWSLPEGSRPCDVACPQDCELSPWSTWSSCSASCGLHAKRTRTKRVLTLPVNG 352

*********************************************:*********:****

Glean3_19739 GRPCAQETDENGLITQYSPCMDIDPCYTYQWVASAWSECQVIGSNCGKGLQTREVYCGR**N** 383

Glean3_25454 GQPCSQETDENGMYISSS--------------------RQVLAI---------------- 376

*:**:*******: . * **:.

Glean3_19739 **DGLEAEDGMCLLDFTQPSPTPTQR**CYIPCGGDCLLSEWSEFGPCQSNCGTESLRNYCRMR 443

Glean3_25454 ------------------------------------------------------------

Glean3_19739 KRDIVGVSMTESLSELCPHIADSDLHEFLLCGLQSSTYSWTFGPWTTCTLAEGLKCGDEG 503

Glean3_25454 ------------------------------------------------------------

Glean3_19739 KQSRAAICLR**DDNVQVADLFCEPLYPSQAGPLEER**PCNVQCSIDCEVTYWSNWTSCSQAC 563

Glean3_25454 ------------------------------------------------------------

Glean3_19739 GQGERTRTR**AITISPIQGGR**ACPLLNDTQMCFERSCDLIEWDISEWRPCLPTDLTTNCGA 623

Glean3_25454 ------------------------------------------------------------

Glean3_19739 GTQSRNISCPVGAENESLCQRRKPKPALTQACHLPCQGECVYSEWSAFTSCSQPCVNAEK 683

Glean3_25454 ------------------------------------------------------------

Glean3_19739 SRSR**IVIRPDAELQPCTPLR**QTETCSQADCLASVYGLATGEWSTCRPIQGECGRGKFQYV 743

Glean3_25454 ------------------------------------------------------------

Glean3_19739 LYRLVQI 750

Glean3_25454 -------

Clustal alignment of entries Glean3:19739 (Sp-Thsd7b) and Glean3:25454 (Sp-Thsd7b_2). Peptides sequenced by MS/MS are in bold red. A possible microheterogeneity is shaded yellow.
